# A Novel Virus of *Flaviviridae* Associated with Sexual Precocity in *Macrobrachium rosenbergii*

**DOI:** 10.1128/mSystems.00003-21

**Published:** 2021-06-08

**Authors:** Xuan Dong, Guohao Wang, Tao Hu, Juan Li, Chen Li, Zhi Cao, Mang Shi, Yiting Wang, Peizhuo Zou, Jipeng Song, Wen Gao, Fanzeng Meng, Guoliang Yang, Kathy F. J. Tang, Cixiu Li, Weifeng Shi, Jie Huang

**Affiliations:** a Yellow Sea Fisheries Research Institute, Chinese Academy of Fishery Sciences, Laboratory for Marine Fisheries Science and Food Production Processes, Pilot National Laboratory for Marine Science and Technology (Qingdao), Key Laboratory of Maricultural Organism Disease Control, Ministry of Agriculture and Rural Affairs, Qingdao Key Laboratory of Mariculture Epidemiology and Biosecurity, Qingdao, China; b Key Laboratory of Etiology and Epidemiology of Emerging Infectious Diseases in Universities of Shandong, Shandong First Medical University & Shandong Academy of Medical Sciences, Tai’an, China; c Shanghai Ocean University, Shanghai, China; d The Center for Infection & Immunity Study, School of Medicine, Sun Yat-sen University, Guangzhou, China; e Dalian Ocean University, Dalian, China; f Huzhou University, Huzhou, China; g School of Public Health, Shandong First Medical University & Shandong Academy of Medical Sciences, Tai’an, China; h Network of Aquaculture Centres in Asia-Pacific, Bangkok, Thailand; Cornell University

**Keywords:** *Flaviviridae*, *Macrobrachium rosenbergii*, precocity, *Crustaflavivirus infeprecoquis* gen. nov., sp. nov.

## Abstract

Since 2010, sexual precocity, a typical sign of the iron prawn syndrome (IPS), resulting in the reduced size of farmed giant freshwater prawns *Macrobrachium rosenbergii*, has caused substantial production losses. However, the cause of IPS was not clear. We ran tests for eight major shrimp pathogens, but none were detected from IPS-affected prawns. We performed the histopathological examination of tissues and identified an eosinophilic inclusion in the perinuclear cytoplasm of cells in various tissues associated with nervous and endocrinal functions in the compound eyes. A subsequent bioassay with viral extracts of IPS-affected samples reproduced the gross signs of IPS. Metatranscriptomic sequencing identified a novel virus of *Flaviviridae* in all IPS-affected *M. rosenbergii* prawns, which was not found in samples without IPS. This virus contains a positive-sense, single-stranded RNA genome of 12,630 nucleotides (nt). Phylogenetic analysis of the conserved RdRp and NS3 domains showed that it may belong to a new genus between Jingmenvirus and *Flavivirus*. Under transmission electron microscopy (TEM), putative virus particles showed as spherical with a diameter of 40 to 60 nm. *In situ* hybridization found hybridization signals consistent with the histopathology in the compound eyes from IPS-affected *M. rosenbergii*. We provisionally name this virus infectious precocity virus (IPV) and propose the binominal Latin name *Crustaflavivirus infeprecoquis* gen. nov., sp. nov. We developed a nested reverse transcription-PCR diagnostic assay and confirmed that all IPS-affected prawns tested IPV positive but normal prawns tested negative. Collectively, our study revealed a novel virus of *Flaviviridae* associated with sexual precocity in *M. rosenbergii*.

**IMPORTANCE** The iron prawn syndrome (IPS), also described as sexual precocity, results in the reduced size of farmed prawns at harvest and significant economic losses. IPS has been frequently reported in populations of farmed *Macrobrachium rosenbergii* since 2010, but the cause was heretofore unknown. Here, we reported a novel virus identified from prawns with IPS using infection experiments, metatranscriptomic sequencing, and transmission electron microscopy and provisionally named it infectious precocity virus (IPV). Phylogenetic analysis showed that IPV represents a new genus, proposed as *Crustaflavivirus* gen. nov., in the family *Flaviviridae*. This study provides novel insight that a viral infection may cause pathological change and sexual maturation and subsequently affect crustacean growth. Therefore, we call for quarantine inspection of IPV in transboundary trade of live *M. rosenbergii* and enhanced surveillance of IPV in aquaculture in the region and globally.

## INTRODUCTION

The giant freshwater prawn *Macrobrachium rosenbergii* can tolerate a wide range of salinities (0 to 25 ppt) and temperatures (18 to 34°C). It has become an important inland aquaculture species, and the global aquaculture production of *M. rosenbergii* has expanded from 136,415 tonnes in 2000 to 287,326 tonnes in 2017 (1, 2). The cultivation of *M. rosenbergii* has been strengthened in China since 1993. China has now become the largest *M. rosenbergii* production country in the world. The pond area is approximately 30,000 ha, and the production reached 139,609 tonnes in 2019 (3). However, the domestic producers have experienced substantial economic losses since 2010, resulting from the so-called iron prawn syndrome (IPS), which is characterized by precocity-associated growth retardation (4, 5). The affected female prawns show sexual precocity, i.e., carrying a brood of eggs on the abdomen, and the affected males have two elongated front claws (2nd pereiopod) but with a much smaller body size than normal prawns (6). Even though there is no significant mortality associated with IPS, the production of affected prawns is reduced by more than 50% as a result of stunted growth. After a decade of IPS outbreak, the cause of IPS remains unknown (5). Healthy postlarvae stocked in IPS-affected ponds develop IPS, which suggests that IPS is contagious.

Currently, the *Flaviviridae* includes four classified genera, *Flavivirus*, *Hepacivirus*, *Pegivirus*, and *Pestivirus* (7), and unclassified clusters, such as Jingmenvirus. Viruses in the genus *Flavivirus* are enveloped, spherical, and 40 to 60 nm in diameter (8), with genomes consisting of a single-stranded, positive-sense RNA approximately 9 to 13 kilonucleotides in length that encodes a single polyprotein. The polyprotein coding region is flanked by 5′ and 3′ untranslated regions (UTRs) with lengths of ∼100 and 400 to 700 nt, respectively (9). The large (approximately 3,500 amino acids [aa]) polyprotein is co- and posttranslationally cleaved to generate three structural proteins (capsid [C], premembrane [prM], and envelope [E]) and seven nonstructural proteins (NS1, NS2A, NS2B, NS3, NS4A, NS4B, and NS5). Currently, more than 50 species of flaviviruses (FVs) have been identified (7); they have been empirically divided into three groups based on their hosts. The first group includes dual-host FVs transmitted between invertebrate vectors (hematophagous arthropods such as mosquitoes and ticks) and vertebrates. The other two groups have no known vectors; they are either vertebrate specific (VSFVs) or insect specific (ISFVs). ISFVs can be further divided into two distinct types based on their phylogenies. Type I is dual host-associated ISFVs (dISFVs), and type II is classical ISFVs (cISFVs) (10).

Recently, a number of novel FVs have been identified from different hosts due to the wide use of metagenomic sequencing, and some of them have been found to be distant from the known groups. For example, a tick-borne segmented Jingmen tick virus (JMTV) has been reported, and two nonstructural proteins from segments of JMTV are related to NS3 and NS5 of *Flavivirus* (11). Subsequently, several other segmented JMTV-like viruses have been described and tentatively classified as the Jingmenvirus group (12–14). In addition, several FVs also have been identified from marine crustaceans, including Crangon crangon flavivirus (CcFv) from a decapod host and Gammarus chevreuxi flavivirus (GcFV) and Gammarus pulex flavivirus (GpFV) from wild-caught malacostracan crustaceans (15). Phylogenetic analysis shows that the crustacean group is more closely related to the terrestrial vector-borne FVs than the cISFVs (15).

In the present study, we sampled *M. rosenbergii* presenting with IPS from farms in Jiangsu Province, China, during 2018 to 2020. Based on results from molecular diagnostics, histopathological examination, laboratory challenge experiments, metatranscriptomic sequencing, phylogenetic analysis, transmission electron microscopy (TEM), and *in situ* hybridization, we identified a novel member of the family *Flaviviridae*, which is associated with IPS and was tentatively named infectious precocity virus (IPV). We have also developed a nested reverse transcription-PCR (RT-PCR) assay specific for IPV as a diagnostic tool.

## RESULTS

### Molecular diagnostics for common shrimp/prawn pathogens.

To discover the potential causative agent, nine IPS-affected *M. rosenbergii* and three normal *M. rosenbergii* prawns were collected from Jiangsu Province, China, in 2018. Diagnostic PCR or RT-PCR was performed to detect eight known shrimp pathogens, including infectious hypodermal and hematopoietic necrosis virus (IHHNV), *Enterocytozoon hepatopenaei* (EHP), acute hepatopancreatic necrosis disease-causing *Vibrio* (*V*_AHPND_), white spot syndrome virus (WSSV), yellow head virus genotype 1 (YHV-1), infectious myonecrosis virus (IMNV), decapod iridescent virus 1 (DIV1), and Taura syndrome virus (TSV). However, none of these pathogens was identified in IPS-affected prawns (see Fig. S1 in the supplemental material).

10.1128/mSystems.00003-21.1 FIG S1 Electrophoretogram of molecular detection of the eight common pathogens. M, molecular marker; O, black control; N, negative control; P, positive control; 1 to 13, samples. S1, the first step PCR amplicon of nested PCR for each pathogen. S2, the second step PCR amplicon of the nested PCR for each pathogen. Download FIG S1, PDF file, 0.2 MB. Copyright © 2021 Dong et al. 2021 Dong et al. https://creativecommons.org/licenses/by/4.0/ This content is distributed under the terms of the Creative Commons Attribution 4.0 International license.

### Histopathological examination of IPS-affected *M. rosenbergii*.

**FIG 1:**
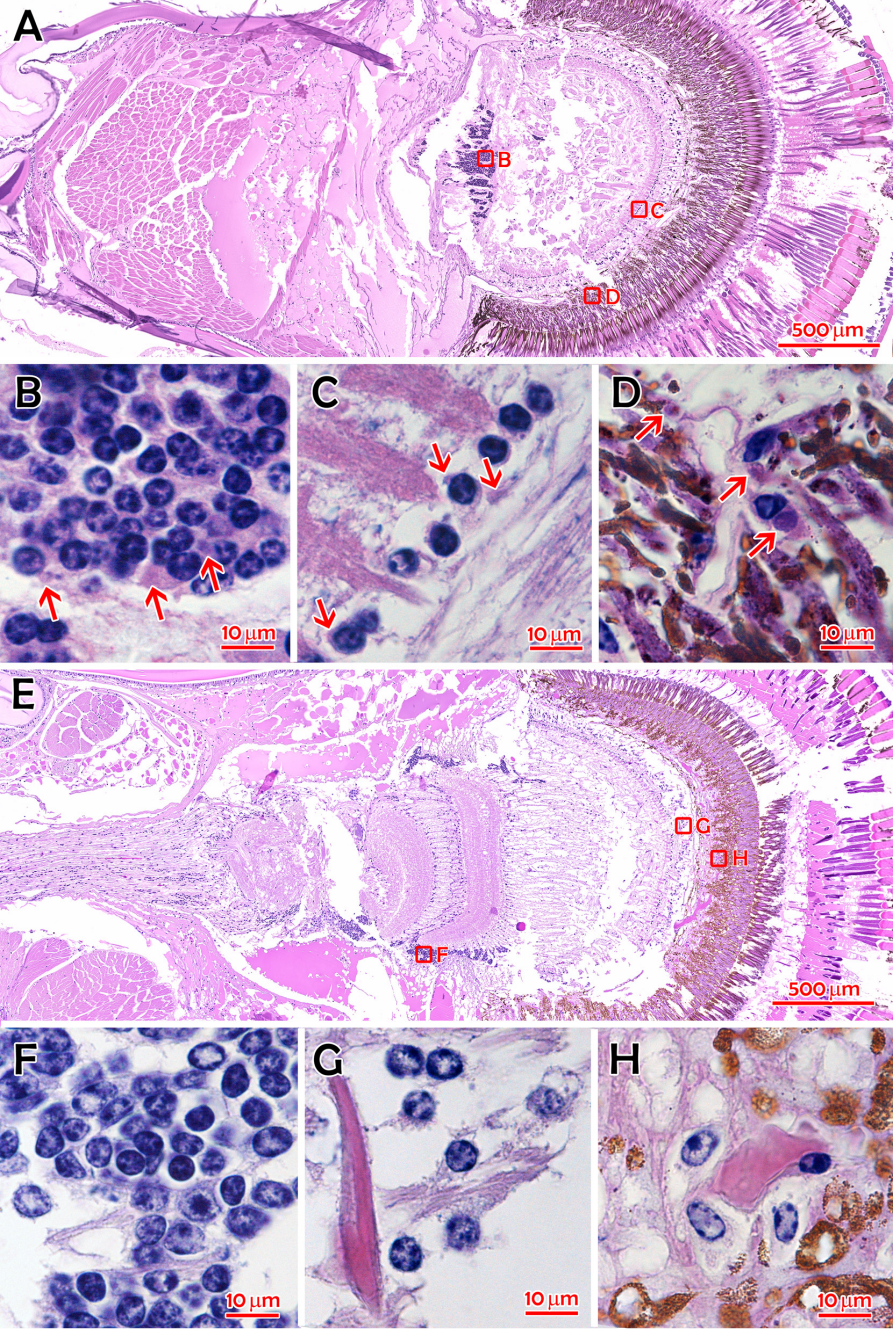
H&E-stained histological sections of *M. rosenbergii* tissues. (A) The overall view of a compound eye of *M. rosenbergii* 0929036 with IPS. (B) Globuli cells in the hemiellipsoid body. (C) Cells in the lamina ganglionaris. (D) Cells in the fasciculated zone. (E) The overall view of a compound eye of *M. rosenbergii* 0929006 without IPS. (F) Globuli cells in the hemiellipsoid body. (G) Cells in the lamina ganglionaris. (H) Cells in the fasciculated zone. Red arrows indicate cytoplasmic inclusions. Bar in panels A and E, 500 μm; bar in panels B, C, D, F, G, and H, 10 μm.

To identify the histopathological feature, we cautiously examined 20 valid hematoxylin and eosin (H&E)-stained tissue sections from 43 *M. rosenbergii* individuals affected with IPS or challenged with the viral extracts compared with 12 sections from 18 normal or control prawns (Fig. 1 and Fig. S2, from farm sample 0929036 versus 0929006 and challenge study sample 0821007 versus 0821006, respectively). Tissues in the compound eyes of the IPS-affected prawns might show different levels of necrosis (Fig. 1A) than those of normal prawns at the lower magnification view. At high magnification, presumed eosinophilic inclusions were observed in the perinuclear cytoplasm of some neurosecretory cells in the organ of Bellonci (the so-called sensory pore X organ) and globuli cells of the hemiellipsoid body (Fig. 1B). They were also seen in cells of various tissues associated with nervous and endocrinal functions, including the lamina ganglionaris (Fig. 1C), the fasciculated zone (Fig. 1D), the onion body (Fig. S2B), and the sinus gland (Fig. S2C). As the lamina ganglionaris and ganglia tissues had extensive eosinophilic and granular cytoplasm interfering with identifying the cytoplasmic eosinophilic inclusions, only the inclusions with a round or elliptical shape and a distinct edge adjacent to the nucleus were distinguishable. In contrast, no similar histopathological characters were observed in normal prawns (Fig. 1E to H and Fig. S2E to H).

10.1128/mSystems.00003-21.2 FIG S2 H&E-stained histological sections of tissues of *M. rosenbergii*. (A) The overall view of a compound eye of *M. rosenbergii* (0821007) with IPS. (B) The magnified onion body with eosinophilic inclusions and reduced membrane layers. (C) Cells in the sinus gland area with inclusions. (D) Cells in the cortical glia. (E) The overall view of a compound eye of *M. rosenbergii* (0821006) without IPS. (F) The magnified onion body. (G) Cells in the sinus gland area. (H) Cells in the cortical glia. Red arrows indicate the eosinophilic inclusion bodies. Bar in panels A and E, 100 μm; bar in panels B, C, D, F, G, and H, 20 μm. Download FIG S2, JPG file, 2.0 MB. Copyright © 2021 Dong et al. 2021 Dong et al. https://creativecommons.org/licenses/by/4.0/ This content is distributed under the terms of the Creative Commons Attribution 4.0 International license.

### Laboratory challenge with viral extracts from IPS-affected prawns.

**FIG 2:**
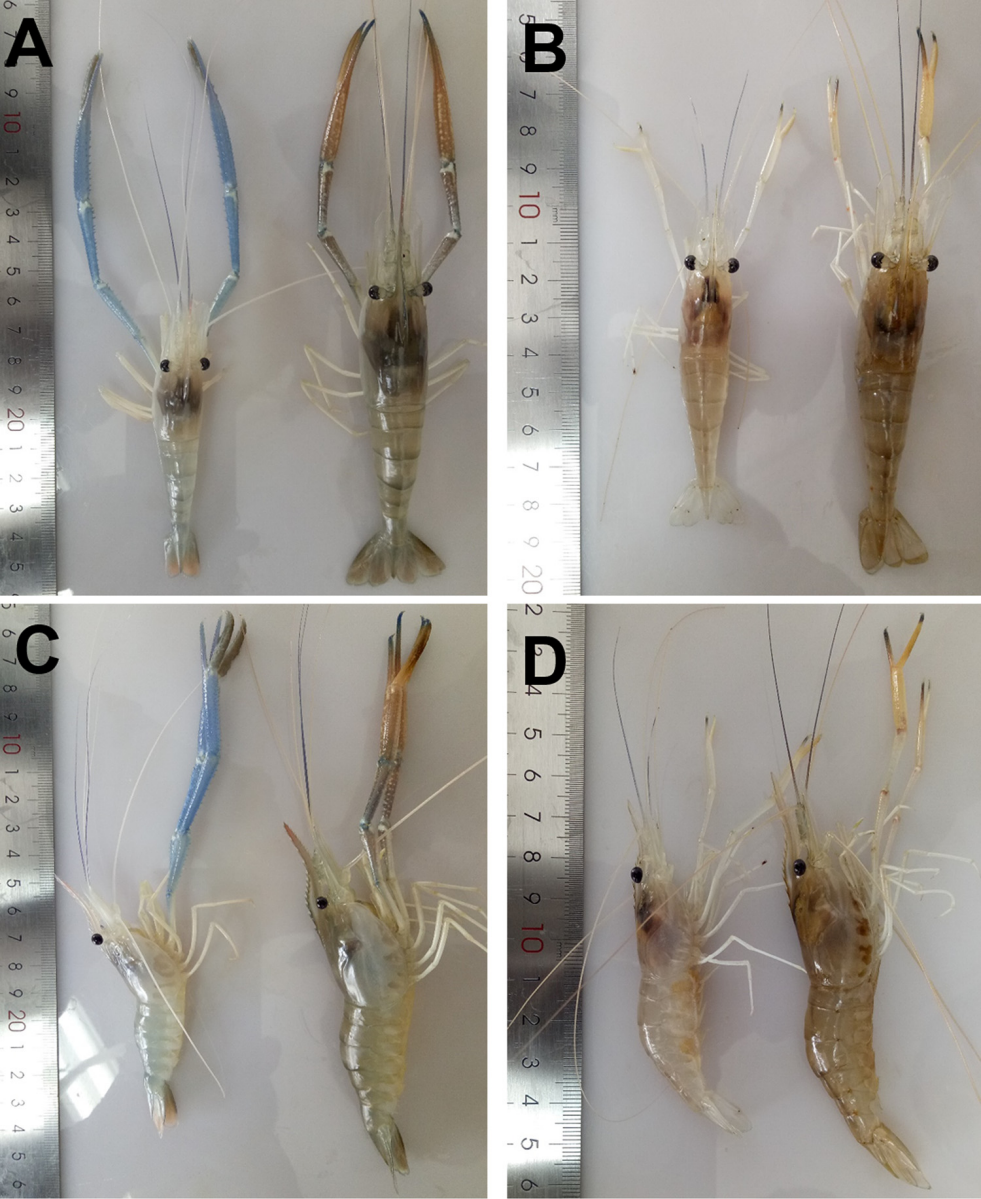
Gross signs of *M. rosenbergii* challenged with IPV preparation. (A and C, left) An infected male *M. rosenbergii*. (A and C, right) A control male. (B and D, left) An infected female *M. rosenbergii*. (B and D, right) A control female.

To determine whether the IPS is caused by an infectious agent, healthy *M. rosenbergii* postlarvae were immersed with filterable viral preparation of the IPS-affected prawns. The infected males exhibited gross signs of IPS (Fig. 2A and C, left) during the 22nd to 25th weeks postinfection, and the claws were blue and longer relative to body length than those of the males in the control group (Fig. 2A and C, right). The challenged females (Fig. 2B and D, left) also exhibited distinct gross signs of IPS, including growth cessation and sexual precocity, which were not observed in the control group (Fig. 2B and D, right). Regarding the body length, the two groups had similar growth until 20 weeks postinfection; however, the infected prawns were significantly (*P* < 0.05) smaller than normal prawns in the control group after the 22nd week postinfection (Fig. S3).

10.1128/mSystems.00003-21.3 FIG S3 Mean body lengths of *M. rosenbergii* in the infected and control groups. Different lowercase superscript letters indicate that the difference is statistically significant within a column (*P* < 0.05). Download FIG S3, TIF file, 0.07 MB. Copyright © 2021 Dong et al. 2021 Dong et al. https://creativecommons.org/licenses/by/4.0/ This content is distributed under the terms of the Creative Commons Attribution 4.0 International license.

### Metatranscriptomic sequencing.

To identify the potential causative agent, metatranscriptomic sequencing was performed for IPS-affected prawns collected from diseased farms (DF1, DF2, and DF3), the viral preparation (DP) of the sample DF0 from farmed prawns with IPS, IPS-affected prawns from the infected groups (DC1, DC2, DC3, and DC4), the normal preparation (NP) of the IPS-free prawns NF0, and prawns from the control group in the challenge study (NC1 and NC2) (Table 1 and Table S1). Viral reads were identified in the samples with IPS, including *Dicistroviridae*, *Flaviviridae*, hepe-like, *Hepeviridae*, levi-like, narna-like, noda-like, partiti-like, *Parvoviridae*, *Phenuiviridae*, picorna-like, qinvirus-like, *Siphoviridae*, and tombus-like viruses (Table 1). Picorna-like viruses were found in five of the eight samples with IPS, and reads of picorna-like viruses were also identified in all prawns without IPS. However, reads associated with *Flaviviridae* were identified in all of the IPS-affected *M. rosenbergii*, whereas they were not found in prawns without IPS (Table 1). These results suggested that IPS was likely associated with a flavivirus, and it was provisionally named infectious precocity virus (IPV).

**TABLE 1:**
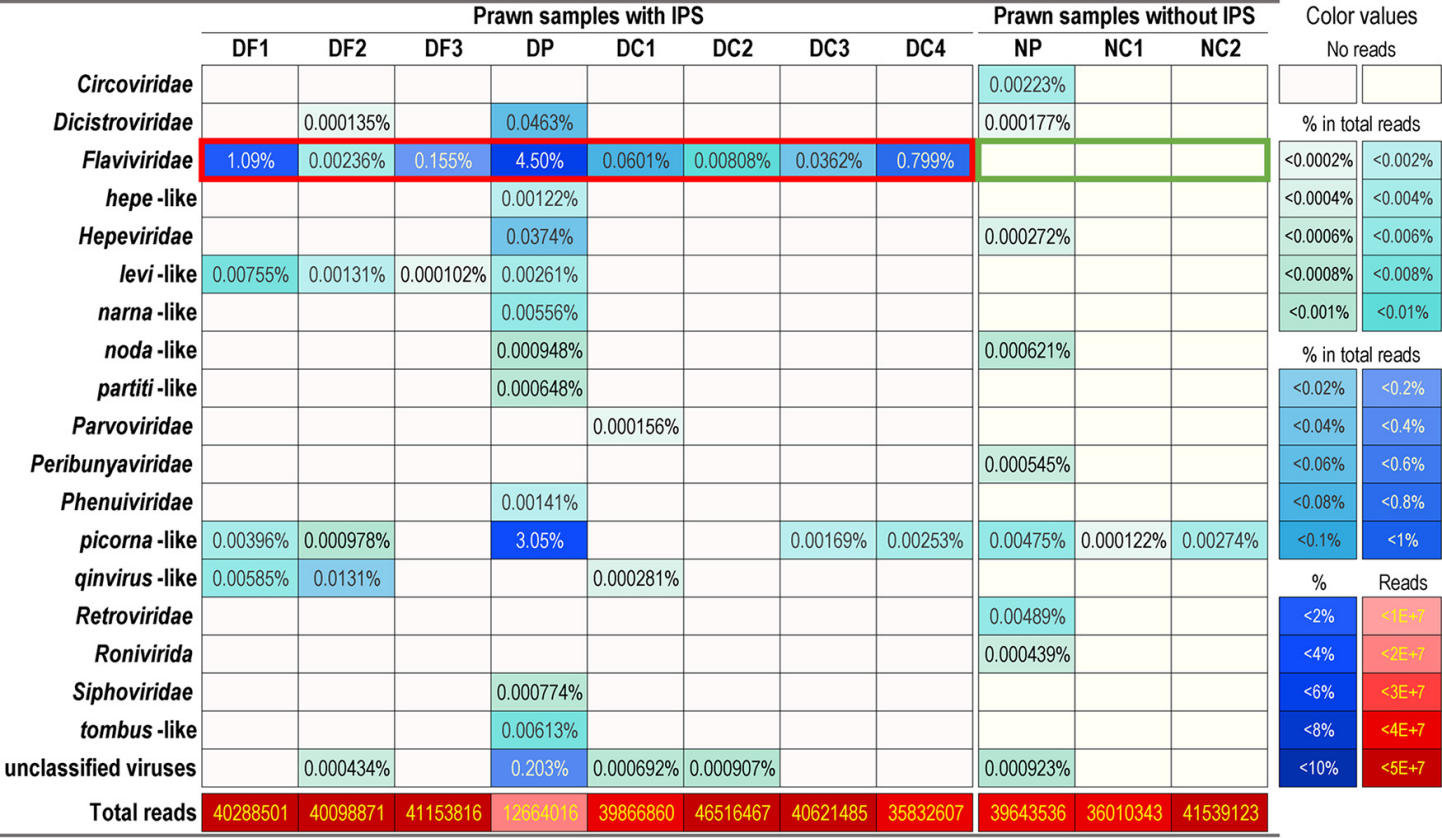
Viral contigs identified from transcriptomic sequencing^*a*^

a Coverage, >40%; identity, >20%; length of contig, >1,000 nt. DF, sample from diseased farm with IPS; DP, viral preparation of prawns from the diseased farm with IPS; DC, diseased sample from the challenge study with the viral preparation; NF, sample from normal farm without IPS; NP, normal preparation from prawns without IPS; NC, sample from challenge study with normal preparation; IPS, iron prawn syndrome. Red and green boxes indicate the full identity between reads and disease status. The sample information is listed in Table S1.

10.1128/mSystems.00003-21.7 TABLE S1 Information of sample marks mentioned in the paper. Download Table S1, PDF file, 0.1 MB. Copyright © 2021 Dong et al. 2021 Dong et al. https://creativecommons.org/licenses/by/4.0/ This content is distributed under the terms of the Creative Commons Attribution 4.0 International license.

### Molecular characterizations of the IPV genome.

**FIG 3:**
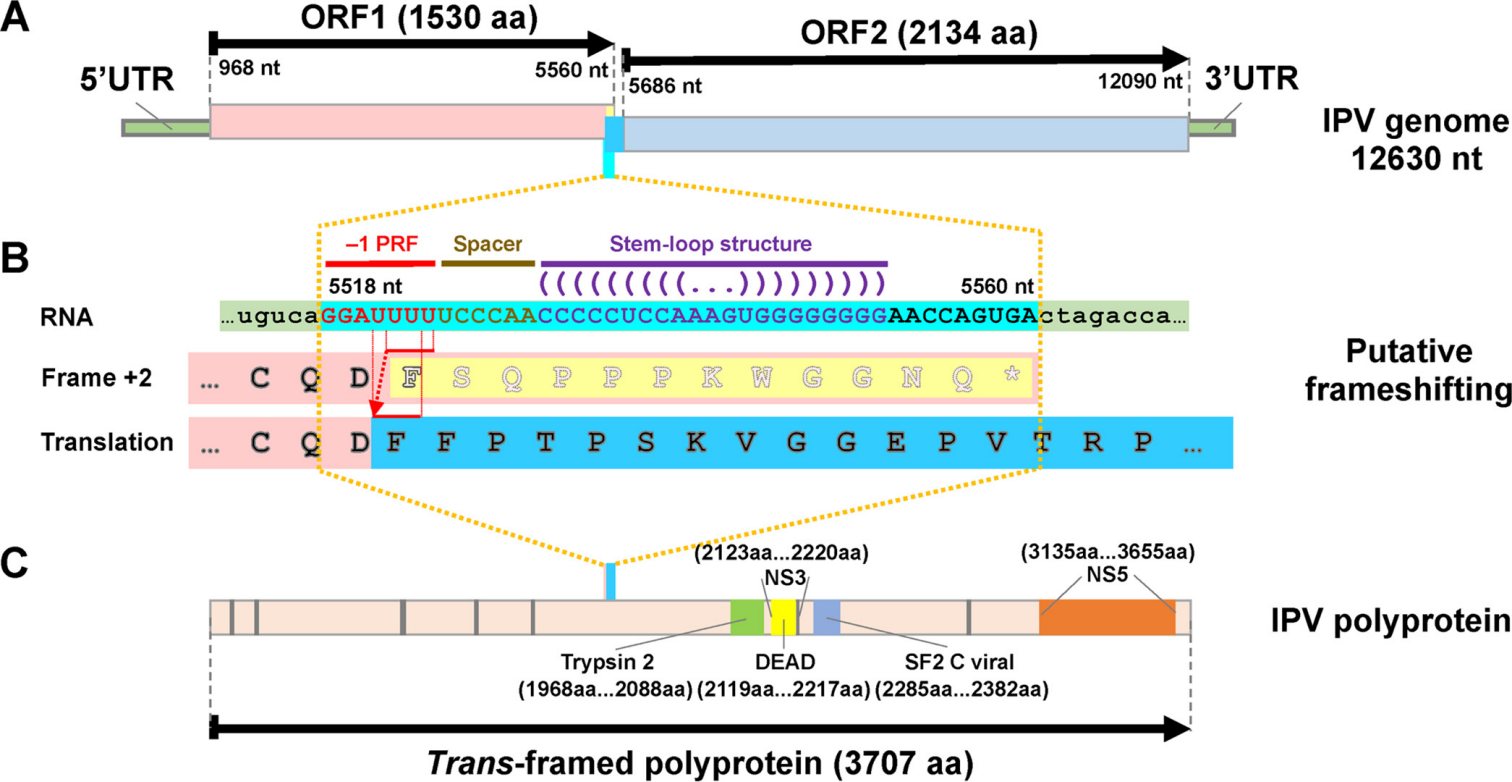
Molecular characterizations of the IPV genome. (A) The full-length genome of IPV with 2 predicted ORFs, 5′ and 3′ UTRs, and potential translational changes caused by the putative frameshifting are indicated. The light yellow block represents the frameshift-off region, taken over by the sky-blue block representing the frameshift-on region. The aqua column indicates the RNA sequence region, which is zoomed out in panel B. (B) The putative frameshifting process. Nucleotide-level details of the aqua column of panel A are presented with the capital RNA sequence with aqua background, in which red letters indicate the potential −1 programmed ribosomal frameshifting (−1 PRF), coffee letters indicate 6-nt spacer, and dark magenta letters indicate the potential stem-loop structure. The red arrow with a dotted line indicates the putative frameshifting. The baby pink background indicates the last fragment of ORF1, in which the hollow letters with a light yellow background indicate the frameshift-off amino acid sequence. The black letters with a sky blue background indicate the frameshift-on amino acid sequence. (C) The predicted conserved domains in the polyprotein.

The complete genome of IPV was determined to be 12,630 nt, including a poly(A) tail at the 3′ end (Fig. 3A), based on a combination of sequencing results from (i) high-throughput RNA sequencing and remapping; (ii) RT-PCR in conjunction with amplicon sequencing; and (iii) 5′ and 3′ rapid amplification cDNA ends (RACE). Using the IPV genome as a query, the BLASTn search failed to find any significant similarity against the GenBank nucleotide database. However, the BLASTx search found significant hits of NS5-like proteins of several flaviviruses, including Wuhan aphid virus 2 (WHAV2), Tamana bat virus (TABV), Yanggou tick virus (YGTV), Wuhan flea virus (WHFV), southern pygmy squid flavivirus (StPSFV), and Saint Louis encephalitis virus (SLEV), although the identities were very low (25 to 30%). Similar identities (26 to 28%) were also found in the NS3 proteins of three flaviviruses: TABV, StPSFV, and SLEV.

The IPV genome was predicted to contain two separate open reading frames (ORFs), ORF1 (1,530 aa) and ORF2 (2,134 aa), with a short intergenic region (125 nt) from positions 5561 to 5685 (Fig. 3A). The remaining untranslated regions (UTRs) consisted of 967 nt and 540 nt at the 5′ and 3′ ends, respectively. Almost all flaviviruses encode one polyprotein, which is produced using the −1 programmed ribosomal frameshifting (−1 PRF), including marine flaviviruses (15–18). Similarly, we found a potential −1 PRF site at positions 5518 to 5524 (Fig. 3B) in the IPV genome, producing a single *trans*-framed polyprotein of 3,707 aa (Fig. 3C), and the slippage heptanucleotide that makes up the site as G_GAU_UUU. A 6-nt spacer region separated this site from a potential stem-loop structure containing 21 nt (positions 5531 to 5551) (Fig. 3B).

In the polyprotein of IPV, we identified several flavivirus-related conserved domains using the Conserved Domain Search (CD-R) (Fig. 3C), including the flavivirus RNA-directed RNA polymerase (Flavi_NS5; pfam00972; E value, ≤1.19E−18; aa positions 3135 to 3655); the flavivirus DEAD domain (Flavi_DEAD; pfam07652; E value, ≤2.80E−12; aa positions 2119 to 2217); the trypsin-like peptidase domain (Trypsin_2; pfam13365; E value, ≤2.85E−4; aa positions 1968 to 2088); the DEXH-box helicase domain of NS3 protease-helicase (DEXHc_viral_Ns3; cd17931; E value, ≤9.64E−11; aa positions 2123 to 2220); and the C-terminal helicase domain of viral helicase (SF2_C_viral; cl18806; E value, ≤1.68E−3; aa positions 2285 to 2382).

### Phylogenetic analysis of the novel IPV.

**FIG 4:**
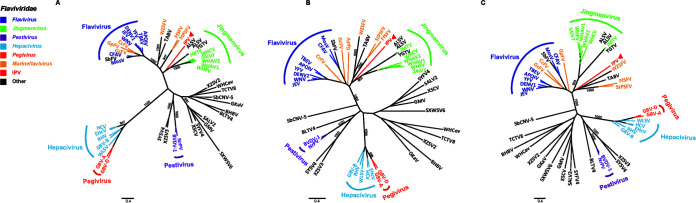
Unrooted phylogenetic trees of the RdRp and NS3 proteins of representative flaviviruses. (A) The RdRp phylogenetic tree was estimated with the sequence alignment trimmed using the heuristic automated1 mode, LG+I+G was employed as the amino acid substitution model, and trees were evaluated by 1,000 bootstrap replicates. (B) The NS3 phylogenetic tree was estimated with the sequence alignment trimmed using the heuristic automated1 mode, RtREV+I+G was employed as the amino acid substitution model, and trees were evaluated by 1,000 bootstrap replicates. (C) The NS3 phylogenetic tree was estimated with the sequence alignment trimmed using the gappyout mode, RtREV+I+G was employed as the amino acid substitution model, and trees were evaluated by 1,000 bootstrap replicates. IPV is highlighted with a red triangle. Abbreviations: Apoi virus (APOIV), cell fusing agent virus (CFAV), dengue virus 1 (DENV1), Japanese encephalitis virus (JEV), mosquito flavivirus K928 (MosV), tick-borne encephalitis virus (TBEV), West Nile virus (WNV), yellow fever virus (YFV), equine hepacivirus (EHcV), hepatitis C virus (HCV), hepatitis GB virus B (GBV-B), rodent hepacivirus (RHV), Wenling shark virus (WLSV), Jingmen tick virus (JMTV), Shuangao insect virus 7 (SAIV7), Wuhan aphid virus 1 (WHAV1), Wuhan aphid virus 2 (WHAV2), Wuhan cricket virus (WHCV), Wuhan flea virus (WHFV), Crangon crangon flavivirus (CcFV), firefly squid flavivirus (FfSFV), Gammarus chevreuxi flavivirus (GcFV), Gammarus pulex flavivirus (GpFV), southern pygmy squid flavivirus (StPSFV), Wenzhou shark flavivirus (WZSFV), pegivirus A (GBV-A), pegivirus B (GBV-D), bovine viral diarrhea virus 1 (BVDV-1), Norway rat pestivirus (NrPV), Alongshan virus (ALSV), Beihai barnacle viurs 1 (BHBV), bole tick virus 4 (BLTV4), Gamboa mosquito virus (GMV), Gentian Kobu-sho-associated virus (GKaV), Sabethes flavivirus (SbFV), Sanxia water strider virus 6 (SXWSV6), Shayang fly virus 4 (SYFV4), Shayang spider virus 4 (SYSV4), Shuangao lacewing virus 2 (SALV2), soybean cyst nematode virus 5 (SbCNV-5), Tacheng tick virus 8 (TCTV8), Tamana bat virus (TABV), Wuhan centipede virus (WHCev), Xingshan cricket virus (XSCV), Xinzhou spider virus 2 (XZSV2), Xinzhou spider virus 3 (XZSV3), and Yanggou tick virus (YGTV).

To determine the taxonomic classification of the IPV, phylogenetic analysis of the conserved domains of the RNA-dependent RNA polymerase (RdRp) (aa positions 3100 to 3700) and NS3 (aa positions 1850 to 2450) proteins was performed using the maximum likelihood method. Results from phylogenetic analyses of the RdRp proteins using different parameters and methods were highly similar (Fig. 4A), and the major groupings of known viruses in the RdRp phylogenetic trees were consistent with those previously reported (12); the IPV fell in the basal position of the Jingmenvirus group (Fig. 4A). However, when different Trimal parameters were employed, different tree topologies of the NS3 proteins were obtained (Fig. 4B and C). In one scenario, IPV fell in the basal position of the Jingmenvirus group (Fig. 4B). All the alignments trimmed using the automated1 mode in Trimal supported this topology. In another scenario, the IPV fell in the basal position of the group, including some marine flaviviruses and TABV (Fig. 4C). This topology was supported by the alignments trimmed using the gappyout mode in Trimal. Therefore, IPV was distant from all of the known genera of the family *Flaviviridae* and could be proposed as a new genus: *Crustaflavivirus* gen. nov., i.e., a virus genus first identified from crustacean under *Flaviviridae*. The recommended Latin name of the virus was *Crustaflavivirus infeprecoquis* gen. nov., sp. nov. based on the binomial nomenclature for virus species (19), of which the Latin species name *infeprecoquis* means infectious precocity.

### Transmission electron microscopy of IPV.

**FIG 5:**
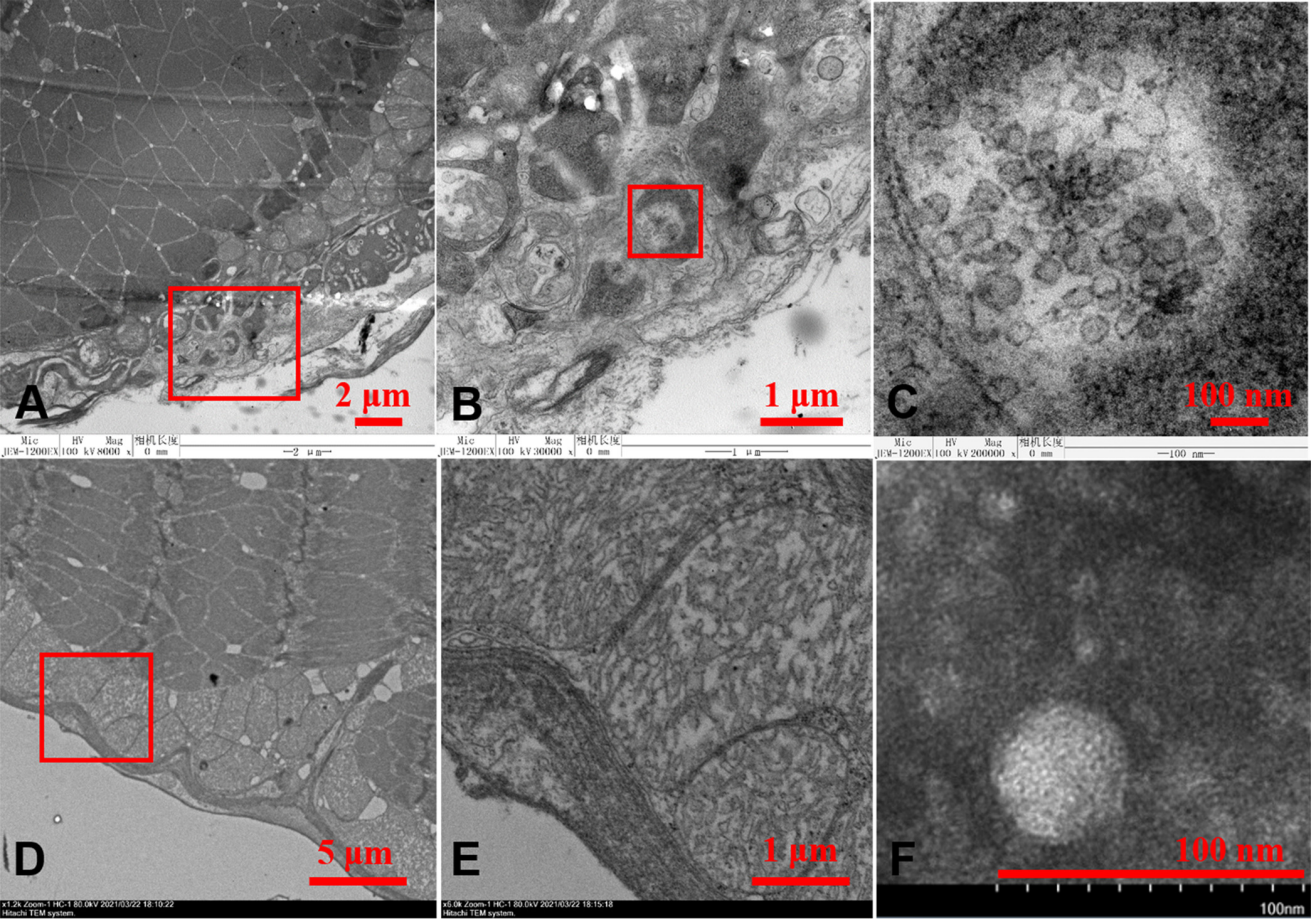
Transmission electron micrographs of the putative IPV particles. (A, B, and C) Eyestalk from IPS-affected *M. rosenbergii*. (B) The magnified view at the red frame of panel A. (C) The magnified view at the red frame of panel B. (D and E) Eyestalk from uninfected *M. rosenbergii*. (E) Purified putative IPV particles stained with 2% phosphotungstic acid (PTA).

Using an electron microscope, we observed agminate virus-like particles in cytoplasmic inclusions on the ultrathin sections of the eyestalk tissue from the IPS-affected prawn (DF5); these particles appeared spherical, approximately 40 to 60 nm in diameter with a higher electron-dense envelop (Fig. 5A to C). The morphology of the putative virus particles revealed similar characteristics with *Flavivirus*. In contrast, no similar virus-like particle was found on ultrathin sections of the eyestalk from the prawn without IPS (Fig. 5D and E). Moreover, transmission electron microscopy of negative-stained IPV particles purified from IPS-affected *M. rosenbergii* by sucrose density gradient ultracentrifugation showed 40- to 60-nm virions, which have a morphology similar to those observed in the eyestalk tissues (Fig. 5F).

### ISH.

**FIG 6:**
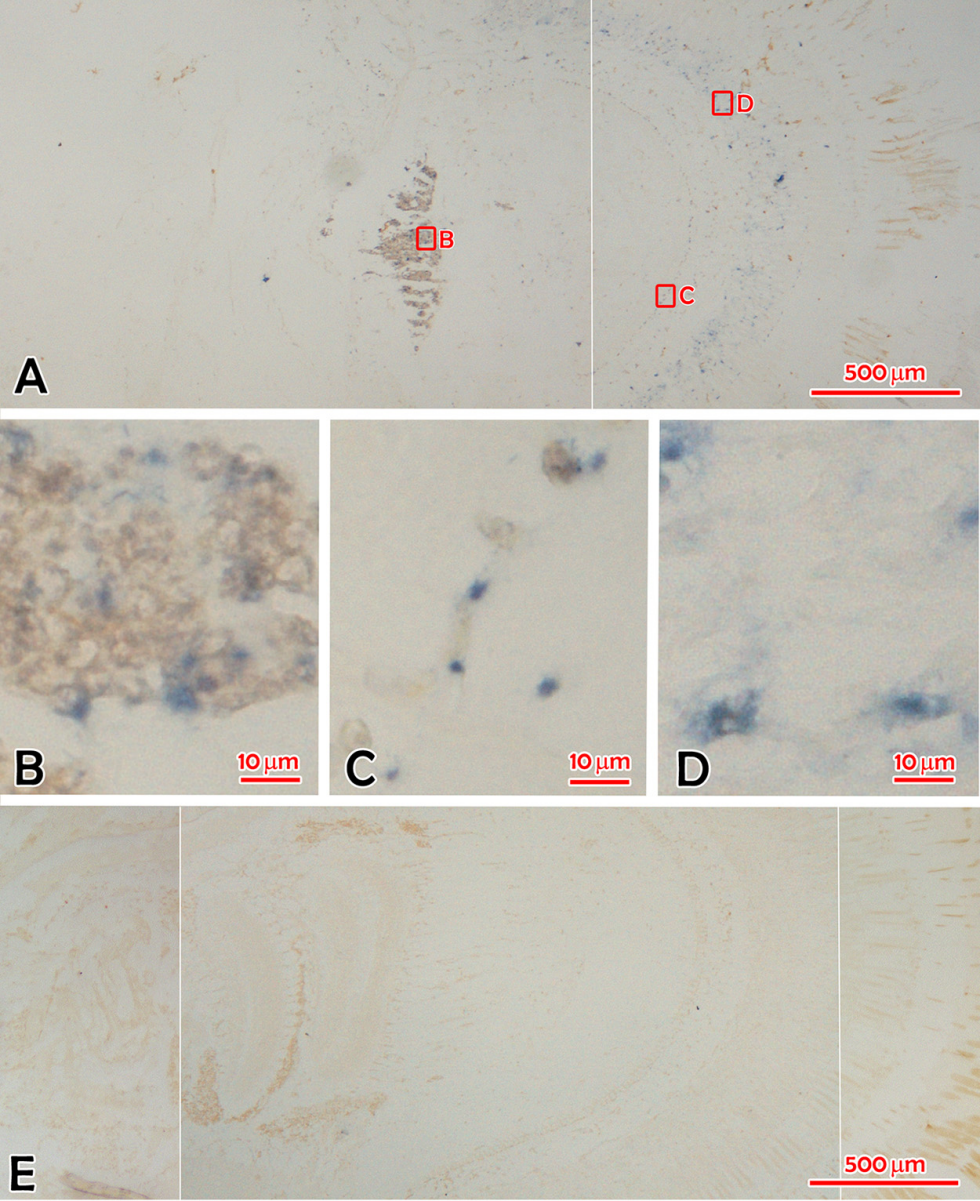
*In situ* hybridization (ISH) micrography of compound eyes from *M. rosenbergii* with and without IPS. (A) The overall view of the blue hybridization signals in the compound eye of IPS-affected *M. rosenbergii* (0929036) shown in Fig. 1A. (B) The hybridization signals in globuli cells in the hemiellipsoid body shown in Fig. 1B. (C) The hybridization signals in cells of the lamina ganglionaris. (D) The hybridization signals in cells of the fasciculated zone. (E) The overall view of the hybridized slide with the compound eye of *M. rosenbergii* (0929006) without IPS shown in Fig. 1E. There was no hybridization signal in the compound eye. Bar, 500 μm (A and E) and 10 μm (B, C, and D).

*In situ* hybridization (ISH) results showed that blue-purple hybridization signals (Fig. 6A, from farm sample 0929036) with the IPV digoxigenin (DIG)-labeled RNA probe presented in the globuli cells (Fig. 6B) in the hemiellipsoid body surrounding the medulla, the rind cells (Fig. 6C) in the lamina ganglionaris, and the primary optic nerve fiber cells (Fig. 6D) in the fasciculated zone of the compound eyes from IPS-affected *M. rosenbergii*. Cells in the onion body (Fig. S4, from farm sample 0929037), the sinus gland, and cortical glia on different slides from the prawns infected with IPV could also show signals of ISH. The ISH signals were identical to the histopathological characters. There was no hybridization signal in the eyes of normal *M. rosenbergii* (Fig. 6E, from farm sample 0929006).

10.1128/mSystems.00003-21.4 FIG S4 *In situ* hybridization (ISH) micrography of the compound eye from *M. rosenbergii* with IPS. (A) The view of the blue hybridization signals in a compound eye of IPS-affected *M. rosenbergii* (0929037). (B) The magnified view at the red frame of panel A. Bar, 20 μm (B) and 100 μm (A). Download FIG S4, PDF file, 0.08 MB. Copyright © 2021 Dong et al. 2021 Dong et al. https://creativecommons.org/licenses/by/4.0/ This content is distributed under the terms of the Creative Commons Attribution 4.0 International license.

### Detection of IPV with nested RT-PCR.

A nested RT-PCR assay specific to IPV was developed, which generated two amplicons of 1,038 bp and 395 bp after the 1st and 2nd step, respectively, in the nested RT-PCR. This assay did not cross-react to Tembusu virus (TMUV), *Macrobrachium rosenbergii* nodavirus (MrNV), yellow head virus genotype 8 (YHV-8), covert mortality nodavirus (CMNV), and the prawn RNA (Fig. S5). We have confirmed the presence of IPV in samples from Table 1 using the nested RT-PCR assay (Fig. S6). A total of 142 *M. rosenbergii* prawns collected from 36 farms were tested using this assay, including 73 (from 18 farms) prawns exhibiting gross signs of IPS, 26 (from 4 farms) prawns without IPS used as negative controls, and 43 prawns collected from 14 farms with unknown IPS status. As expected, all 26 prawns from farms without IPS were negative for IPV; all 73 prawns collected from farms with IPS were positive for IPV after the 2nd step of PCR, with 67 positives after the 1st step of PCR. Among the 43 prawns with unknown IPS status, four prawns were positive for IPV after the 1st step of PCR, and 35 prawns were positive for IPV after the 2nd step of PCR (Table S2).

10.1128/mSystems.00003-21.5 FIG S5 Specificity analysis of the nested RT-PCR method. M, molecular marker; 1, Tembusu virus (TMUV); 2, *Macrobrachium rosenbergii* nodavirus (MrNV); 3, yellow head virus genotype 8 (YHV-8); 4, covert mortality nodavirus (CMNV); 5, healthy *M. rosenbergii*; 6, blank control; 7, IPS-affected *M. rosenbergii*. Download FIG S5, PDF file, 0.07 MB. Copyright © 2021 Dong et al. 2021 Dong et al. https://creativecommons.org/licenses/by/4.0/ This content is distributed under the terms of the Creative Commons Attribution 4.0 International license.

10.1128/mSystems.00003-21.6 FIG S6 Electrophoretogram of IPV nested RT-PCR of samples in Table 1. M, molecular marker; 1, DF1; 2, DF2; 3, DF3; 4, DP; 5, DC1; 6, DC2; 7, DC3; 8, DC4; 9, NP; 10, NC1; 11, NC2; 12, positive control; 13, negative control; 14, blank control. Download FIG S6, PDF file, 0.1 MB. Copyright © 2021 Dong et al. 2021 Dong et al. https://creativecommons.org/licenses/by/4.0/ This content is distributed under the terms of the Creative Commons Attribution 4.0 International license.

10.1128/mSystems.00003-21.8 TABLE S2 Nested RT-PCR detection of IPV in *M. rosenbergii.* Table S2, PDF file, 0.3 MB Copyright © 2021 Dong et al. 2021 Dong et al. https://creativecommons.org/licenses/by/4.0/ This content is distributed under the terms of the Creative Commons Attribution 4.0 International license.

## DISCUSSION

IPS-affected prawns show sexual precocity and reduced size at harvest, resulting in substantial production loss (6). However, the causative agent underlying IPS was previously unknown. In the present study, we identified a novel virus of *Flaviviridae* associated with this syndrome, provisionally named IPV. This virus possesses a typical genomic organization and conserved domains of flaviviruses, including RdRp and NS3. However, the blast search revealed only ∼30% amino acid sequence identity between IPV and the most related flaviviruses. Apart from the two nonstructural proteins, no homology was identified in the structural proteins, which suggested the high genomic divergence and species diversity of flaviviruses. In addition, IPV has a number of notable molecular characterizations. First, the 5′ UTR of IPV was 967 nt in length, much longer than the typical length (∼100 nt) found in FVs (9). Second, a poly(A) tail at the 3′ end is identified, whereas FVs are rarely polyadenylated (10). Third, the −1 PRF site of IPV is identical to four known ISFVs, including Chaoyang virus (CHAOV), Lammi virus (LAMV), Marisma mosquito virus (MMV), and Nanay virus (17, 20). However, the slippage heptanucleotide motif in IPV is G_GAU_UUU, while X_XXY_YYZ presents in the majority of flaviviruses (16, 18).

To determine the phylogenetic position of IPV, we performed a phylogenetic analysis of the RdRp and NS3 protein sequences using different parameters. Consistent with a previous report (12), phylogenies obtained using RdRp and NS3 were not always consistent. Furthermore, phylogenies obtained using different trimming modes also were not always same. Therefore, when the protein sequence identity is low (e.g., ∼30% sequence identity between IPV and the Jingmenvirus and *Flavivirus* in our case), the phylogeny might become sensitive to the alignment method and also the trimming method. However, we propose that it belongs to a new genus of *Flaviviridae*, to be named *Crustaflavivirus* gen. nov.

Crustacean eyes function as an important neuroendocrine system, and eyestalk ablation influences gonadal development (21). Eyestalk ablation is commonly used to accelerate maturation and synchronize spawning in shrimp and prawn hatcheries worldwide (22–24). The maturation of the gonad is regulated by gonad-inhibiting hormone (GIH) secreted from the X-organ–sinus gland (XO–SG) complex of the eyestalk and gonad-stimulating factor (GSF) produced by the brain and thoracic ganglion (22, 25). Histological changes with eosinophilic inclusions and *in situ* hybridization signals in the cells of a variety of tissues in the eyes of IPV-infected *M. rosenbergii* were observed by comparison with the normal tissues of the prawns without IPS. Therefore, it is speculated that sexual precocity is caused by the IPV infection in these tissues of eyes just like the functional ablation of eyestalks. Many dual-host FVs, such as microcephaly-causing Zika virus, spread by daytime-active *Aedes* mosquitoes (26). Mosquito-borne Japanese encephalitis virus (JEV) (27) and tick-borne encephalitis virus (TBEV) (28) are known to infect the central nervous system (CNS) of the vertebrate hosts, but their pathogenic effect on the invertebrate vectors remains unknown. For insect-specific FVs, infected hosts are asymptomatic, although cytopathic effects have been observed in cell cultures (29). Pathological brain lesions in children may cause precocity or early puberty in humans (30). However, we have not found any report on precocity in humans or animals caused by an infectious agent. This study reveals an interesting finding that the IPV infection in the neuroendocrine system of prawns accounts for sexual precocity.

Usually, producers partially harvest several times with sequential stocking of postlarvae during a production cycle. During each partial harvest, large prawns are picked out and a new batch of smaller prawns is added. This operation would likely increase the risk of IPV infection. It was previously impossible to screen postlarval batches prior to stocking, because IPV-infected prawns are asymptomatic until they reach sexual maturity, as shown in our infection experiments, and, therefore, cannot be detected by visual inspection. All of these factors likely resulted in the rapid spread of IPV. The RT-PCR detection assay developed in the present study has proven to be specific and useful for routine diagnosis and monitoring of IPV in prawn stocks and even in pond environments.

*M. rosenbergii* was initially introduced to China from Japan in 1976, with subsequent introductions from Thailand, Myanmar, India, and other countries in the region and was still intermittently imported in recent years. Stunted pond-cultured *M. rosenbergii* with similar signs with IPS has been reported in India and other countries since 2007 (31). In 2010, an extremely large number of postlarval *M. rosenbergii* was imported into China (4, 6). Afterwards, IPS was reported (32). Since 2012, IPS has become a major problem in the prawn farming industry in China (33). From the timeline, it is speculated that the international trade in *M. rosenbergii* facilitated the IPV spread, and the quarantine inspection of IPV for *M. rosenbergii* stock is highly recommended.

Taken together, we described a new virus of *Flaviviridae* from IPS-affected prawns in China. The novel virus had low sequence identity with reported flaviviruses but possessed a typical flavi-like virus genomic organization and conserved domains of *Flaviviridae*. Phylogenetic analysis showed that it was distant from the known or proposed genera. Therefore, we proposed a novel genus named *Crustaflavivirus* gen. nov. and a novel species with the proposed binomial Latin name *Crustaflavivirus infeprecoquis* gen. nov., sp. nov. In particular, analysis of the challenge study showed that infection with IPV caused the clinical signs of sexual precocity associated with stunted prawns, resulting in pathological changes in neurosecretion-related tissues of the compound eyes. The molecular detection revealed that the presence of IPV was associated with the farm cases of IPS. The nested RT-PCR of IPV provides a necessary diagnostic tool for developing an active surveillance program to reveal the epidemiology of IPV infection and investigate the causative agent causing stunted-pond farmed *M. rosenbergii* in countries farming *M. rosenbergii* (31, 34).

## MATERIALS AND METHODS

### Sampling and processing.

In 2018 to 2020, *M. rosenbergii* prawns, presenting with IPS and non-IPS, were collected from farmed ponds in Jiangsu Province, China (see Tables S1 and S2 in the supplemental material). The body lengths of *M. rosenbergii* were measured from the base of eyestalk to telson. Samples of the cephalothoraxes (heads) were preserved in 95% ethanol for metagenomics sequencing and RT-PCR detection. Samples of the cephalothoraxes were fixed in Davidson’s alcohol formalin acetic acid (DAFA) fixative for histopathological and *in situ* hybridization analyses (35). For the TEM study, tissues were preserved in 4% phosphate-buffered glutaraldehyde fixative. Frozen samples were used for the challenge study. Healthy postlarvae of *M. rosenbergii* for the laboratory challenge were purchased from an IPS-free prawn farm in Jiangsu Province. Prawns for IPV RT-PCR analysis were sampled from farms during 2018 to 2020 (Table S2).

### Molecular detection of shrimp pathogens.

Total RNA and DNA were separately extracted from the *M. rosenbergii* samples preserved in 95% ethanol, and the presence of known shrimp pathogens was tested by RT-PCR or PCR methods recommended by the World Organisation for Animal Health (OIE) and published papers (36–39). These shrimp pathogens included WSSV, IHHNV, DIV1, TSV, YHV1, IMNV, *V*_AHPND_, and EHP.

### Viral extracts from *M. rosenbergii*.

Twenty grams of *M. rosenbergii* cephalothoraxes from an IPS-affected farm (DF0) with no known pathogens were homogenized in 100 ml phosphate-buffered saline (PBS). The suspension was clarified by centrifugation at a low speed, and the supernatant was then centrifuged at 8,000 rpm for 30 min at 4°C (CR21GIII; Hitachi, Japan). All of the precipitated dregs were rehomogenized in 100 ml PBS and centrifuged at 8,000 rpm for 30 min at 4°C again. Particles with a sedimentation coefficient larger than 4,650 in the sample, which are equivalent to a size and buoyant density combination around 220 to 310 nm and 1.37 to 1.20 g/cm^3^, were removed from the supernatant. The supernatant was filtered through a 0.22-μm membrane syringe filter to remove bacteria and was used for the challenge study as the viral extract.

### Challenge study with viral extracts.

Healthy *M. rosenbergii* postlarvae (mean body length, 0.6 cm) for the experimental challenge study were acquired from the farm without IPS in Jiangsu Province, China. These prawns were tested negative for the major freshwater prawn and penaeid shrimp pathogens. For the immersion challenge study, the viral extract prepared from IPS-affected prawns was diluted 10^−4^ (vol/vol) in PBS. Healthy *M. rosenbergii* prawns were immersed in a diluted viral solution for 1 h and then transferred to a 9.8-liter tank containing viral solution at a dilution of 10^−6^. The infection group included four biological replicates (with 45 to 50 individuals in each tank). As a negative control, another group of prawns was exposed to PBS only, and the negative group included three biological replicates (with 45 to 50 individuals in each tank). Both groups were held in freshwater at 28 ± 1°C and fed with a pelletized ration for 25 weeks. Prawns were monitored daily for mortality or unusual signs, and the body lengths of individual prawns were measured using pictures taken in shallow water with a ruler. Prawns from each group (the infected group included four biological replicates and the control group included three biological replicates) were sampled at each time point (Table S1). Samples of prawns were fixed in 95% ethanol, 4% phosphate-buffered glutaraldehyde fixative, DAFA fixative, and liquid nitrogen for further studies.

### Viral preparation with differential centrifugation.

Fifteen grams of cephalothoraxes of *M. rosenbergii* from the IPS-affected farm (DF0) and 15 g cephalothoraxes of healthy *M. rosenbergii* from the normal farm without IPS (NF0) were homogenized in 100 ml SM buffer (50 mmol/liter Tris-HCl, 10 mmol/liter MgSO_4_, 100 mmol/liter NaCl, pH 7.5) with 2 ml 4-(2-aminoethyl)benzene sulfonyl fluoride (AEBSF) (Solarbio) (0.35 mmol/liter). The suspension was clarified using centrifugation at a low speed, and the supernatant was then centrifuged at 10,000 × *g* for 30 min at 4°C. All of the precipitated dregs were suspended in 100 ml SM buffer and centrifuged at 8,000 × *g* for 20 min at 4°C again. All supernatants from the above-described centrifugation were mixed and centrifuged at 20,000 × *g* for 20 min at 4°C and then centrifuged with a P50AT rotor at 120,000 × *g* for 4 h at 4°C (CP100WX; Hitachi, Japan). The pellets were collected and used for transcriptomic sequencing.

### Nucleic acid extraction, library preparation, and transcriptome sequencing.

Total RNA of *M. rosenbergii* from IPS-affected farms (DF1, DF2, and DF3), the viral preparation (DP) from DF0, IPS-affected *M. rosenbergii* from the infected group (DC1, DC2, DC3, and DC4), the normal preparation (NP) from NF0, and normal *M. rosenbergii* from the control group in the challenge study (NC1 and NC2) (Table S1) was extracted using the TRIzol reagent (Invitrogen, USA). rRNA was removed using the Epicentre Ribo-zero rRNA removal kit (Epicentre, USA). Sequencing libraries were generated using the rRNA-depleted RNA by NEBNext Ultra directional RNA library prep kit for Illumina (NEB, USA), and then 150-nt paired-end read sequencing of the RNA libraries was conducted using the Illumina HiSeq platform by Novogene (Beijing, China). The raw sequencing reads were adaptor and quality trimmed using the Trimmomatic (40) program embedded in Trinity (41). The clean reads were directly *de novo* assembled using Trinity with default parameter settings. All the assembled contigs were compared against the nonredundant protein database (nr) downloaded from GenBank using BLASTx, with an E value threshold of 1 × 10^−5^. All potential viral contigs were identified and then merged to form longer viral contigs using Bowtie (42) and Geneious (version 11.1.5) (https://www.geneious.com) (43) as previously described (44).

The quality of the contig annotated as a flavivirus was then identified and examined by read mapping, and the results (in sam/bam format) were visualized using Geneious (https://www.geneious.com/). The read-mapping step was iterative in order to extend the genome on both ends. The consensus sequence determined from the final assembly of the mapped reads was used as the newly identified virus genome.

### Viral genome verification and sequence analysis.

To confirm the transcriptome sequencing results, we performed RT-PCR and Sanger sequencing. A set of primer pairs was designed based on the assembled contig (Table S3). Meanwhile, both 5′ and 3′ rapid amplification reactions of cDNA ends (RACE) (Invitrogen) were employed to determine the termini of the obtained viral genome. The open reading frames were predicted using the Open Reading Frame Finder (https://www.ncbi.nlm.nih.gov/orffinder/), and the conserved domains of the predicted polyprotein were analyzed using the Conserved Domain Search (CD-R) available from NCBI.

10.1128/mSystems.00003-21.9 TABLE S3 Nucleotide sequences of PCR primers used in this study. Download Table S3, PDF file, 0.1 MB. Copyright © 2021 Dong et al. 2021 Dong et al. https://creativecommons.org/licenses/by/4.0/ This content is distributed under the terms of the Creative Commons Attribution 4.0 International license.

In addition, reference sequences of the RdRp and NS3 proteins of representative flaviviruses were downloaded from GenBank, and all sequence information are listed in Table S4. Sequence alignment was performed using Mafft (45) with different amino acid substitution models, including Blosum30, Blosum45, and the default Blosum62. The conserved sites were obtained using Trimal (46) with two modes, -automated1 and -gappyout, respectively. The best-fit model of amino acid sequence evolution was determined using Prot-Test 3.4.2 with Akaike information criterion and Bayes information criterion, which were then used in the subsequent phylogenetic analyses. Phylogenetic trees were inferred using the maximum likelihood method (ML) implemented in PhyML version 3.1. Statistical support for the phylogeny was assessed using the approximate likelihood ratio test (aLRT) with a Shimodaira-Hasegawa-like procedure and bootstrapping with 1,000 replicates.

10.1128/mSystems.00003-21.10 TABLE S4 Information of sequences used in Fig. 4. Download Table S4, DOCX file, 0.023 MB. Copyright © 2021 Dong et al. 2021 Dong et al. https://creativecommons.org/licenses/by/4.0/ This content is distributed under the terms of the Creative Commons Attribution 4.0 International license.

### Visualization of IPV.

Ultrathin sections of samples from diseased prawns, infected prawns, and viral preparations were examined under TEM. Small pieces of eyestalk samples in ∼1 mm^3^ of prawns were fixed in TEM fixative at 4°C. Ultrathin sections were prepared on collodion-coated grids by the Medical College of Qingdao University (38). All grids were examined under a JEOL JEM-1200 electron microscope (Jeol Solutions for Innovation, Peabody, MA, USA) and HT7700 (Hitachi, Japan) operating at 80 to 100 kV.

### Viral purification with sucrose density gradient centrifugation.

About 2.5 g eyestalk of *M. rosenbergii* from the IPS-affected farm (DF4) was homogenized in 50 ml SM buffer with 0.5 mmol/liter AEBSF using TissueLyser. Following the procedure described in the section on viral preparation with differential centrifugation, the pellets were resuspended in the SM buffer and loaded onto a prepared density gradient with equal volumes of 18.6%, 28.2%, 37%, 45.3%, and 53% (wt/wt) sucrose and centrifuged with a P40ST rotor at 120,000 × *g* for 5 h at 4°C. The bands collected from the sucrose density gradients were diluted and centrifuged with a P90AT rotor at 120,000 × *g* for 4 h at 4°C, and the pellets were resuspended in SM buffer. The purified virions were dropped on grids and negatively stained with 2% phosphotungstic acid (PTA) (pH 6.5). All the grids were examined under a TEM (HT7700; Hitachi, Japan).

### Synthesis of RNA probe.

The 395-bp amplicon from the 2nd-step RT-PCR was extracted and ligated with PMD18-T vector (TaKaRa). The recombinant plasmid was transformed into TOP10 competent Escherichia coli (TIANGEN). A single clone was selected from Luria-Bertani (LB) agar supplemented with ampicillin (Amp) (Solarbio) and sequenced. Plasmid DNA was extracted from the positive clone. The set of primers F (5′-GTA CCC GGG GAT CCT CTA GAG AT-3′) and R (5′-TAA TAC GAC TCA CTA TAG GGT TGC ATG CCT GCA GGT CGA CGA T-3′) with T7 transposon sequence (underlined) were used to amplify and tail the template of the RNA probe. The reaction was performed in a 20-μl mixture containing 10 μl Premix *Taq* (with 0.5 U *Ex Taq*, 4 nmol deoxynucleoside triphosphate, and 40 nmol Mg^2+^) (TaKaRa), 10 pmol of each primer, and 1 μl DNA template. The amplification was performed with initial denaturation at 95°C for 4 min, followed by 30 cycles of 95°C for 30 s, 58°C for 30 s, and 72°C for 30 s, with a final extension at 72°C for 10 min. The digoxigenin-labeled antisense RNA probe was synthesized with 1 μg template, 2 μl 10× DIG RNA labeling mixture (Roche), 4 μl transcription optimized 5× buffer (200 mmol/liter Tris-HCl, 30 mmol/liter MgCl_2_, 10 mmol/liter spermidine, 50 mmol/liter NaCl, pH 7.9) (Promega), 1 μl T7 RNA polymerase (20 U/μl) (Promega), 2 μl dithiothreitol (100 mmol/liter) (Promega), and 1 μl RNase inhibitor (40 U/μl) (New England Biolabs). The mixture was incubated at 37°C for 2.5 h, placed on ice for 2 min, and then digested with 5 U RNase-free DNase I (Thermo Fisher) at 37°C for 15 min. The probe was purified by a SigmaSpin sequencing reaction clean-up, postreaction clean-up columns (Sigma) kit. NanoDrop 2000 (Thermo Fisher) was used to detect the concentration and quality of the probe, and then we stored the probe at −80°C.

### *In situ* hybridization.

ISH was performed according to the previously published procedure (47, 48), with some modifications. After dewaxing and rehydration, tissue sections were treated with HCl (0.2 mol/liter; 20 min) and proteinase K (20 μg/ml; 30 min; 37°C) (TaKaRa). After washing with phosphate buffer containing Tween 20 (PBST), the slides were prehybridized for 4 h at 42°C in the mixture of 50% formamide, 5× saline citrate (SSC) (Solarbio), 0.1% Tween 20 (Solarbio), 1.9 g/liter citric acid monohydrate, 500 μg/ml tRNA (Sigma), and 50 μg/ml heparin sodium (Solarbio). Hybridization was performed in the same solution mixed with 1 mg/ml DIG-labeled RNA probe at 42°C for 16 h. To detect probes hybridized with viral RNA, we incubated tissue sections with anti-DIG-AP Fab fragments (Roche) for 12 h at 4°C and then stained the hybridization with BCIP (5-bromo-4-chloro-3-indolyl phosphate) and NBT (4-nitroblue tetrazolium chloride) (Roche). The slides were counterstained with Bismarck brown. Healthy prawns served as a negative control by performing the same protocol.

### Development of an IPV-specific nested RT-PCR assay.

Total RNA was extracted from cephalothoraxes tissues using the RNAprep pure tissue kit (TIANGEN). The extracted RNA was reverse-transcribed at 42°C for 45 min and 90°C for 5 min using a PrimeScript II first-strand cDNA synthesis kit (TaKaRa). The 1st step of the nested RT-PCR assay employed the outer primers IPV_F1 (5′-GCA CAC TCC CAA CAC GTT TC-3′) and IPV_R1 (5′-CGC GCG TAA TCT CTA CAC CT-3′). The 1st-step RT-PCR amplified a 1,038-bp fragment from the viral genome. The inner primers for the 2nd-step PCR were IPV_F2 (5′-TCC CTA GGC AGG GGA TAC TG-3′) and IPV_R2 (5′-AGC TAT CCG TGG TGT GGA AC-3′), amplifying a 395-bp fragment. The 1st-step PCR in a 20-μl mixture, containing 10 μl Premix *Taq* (with 0.5 U *Ex Taq*, 4 nmol dNTP, and 40 nmol MgCl_2_) (TaKaRa), 2.5 pmol IPV_F1, 2.5 pmol IPV_R1, and 1 μl template, was initiated at 94°C for 2 min, followed by 30 cycles of 94°C for 30 s, 59°C for 30 s, and 72°C for 65 s, ending with 72°C for 10 min. The 2nd PCR step was performed with the same protocol, except using IPV_F2 and IPV_R2 for the primers, the 1st-step product for the template, and 30 s for the 72°C extension in each cycle. The PCR products were analyzed in a 2% agarose gel containing GeneFinder (Bio-V, China). To test the specificity, we used the samples extracted from healthy *M. rosenbergii* and samples infected with TMUV, MrNV, YHV-8, and CMNV as templates in the nested RT-PCR assay.

### Statistics analysis.

All statistical analyses were performed using the SPSS statistical software package for Windows, version 20.0 (SPSS Inc., Chicago, IL, USA). The body lengths of prawns in different groups were compared by using the *t* test. A *P* value of <0.05 was considered significant.

### Data availability.

The complete genome sequence of IPV has been deposited in GenBank under the accession no. MT084113. The raw data from the metatranscriptomic sequencing analysis of *M. rosenbergii* with and without IPS has been deposited in the NCBI Sequence Read Archive (SRA) database under the BioProject accession number PRJNA675895 (Table S1).
