# Supplementary figures and images for: A Novel Virus of Flaviviridae Associated with Sexual Precocity in Macrobrachium rosenbergii
Source: mSystems. 2021 Jun 8;6(3):e00003-21. doi: 10.1128/mSystems.00003-21 (PMC8269200; doi:10.1128/mSystems.00003-21)

IHHNV-389

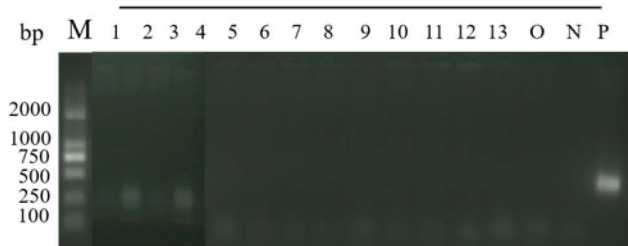

IHHNV-309

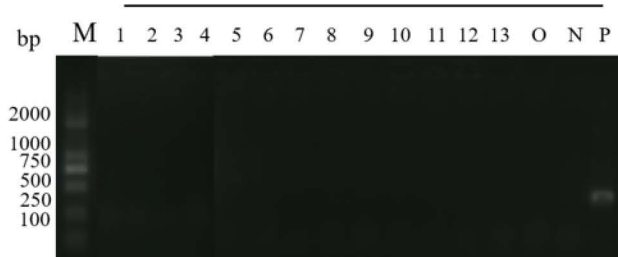

YHV-S1

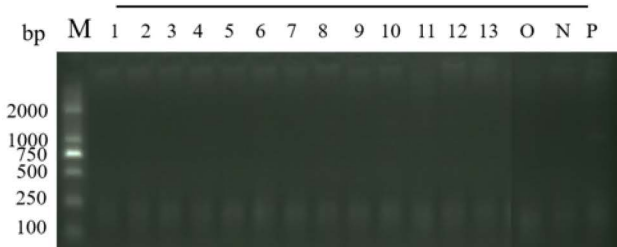

YHV-S2

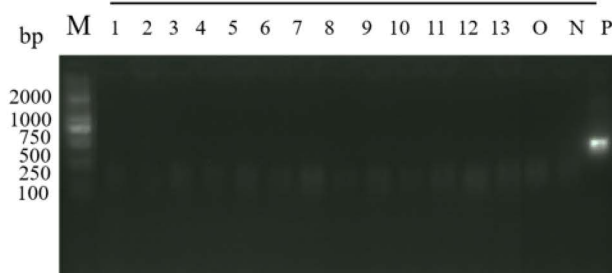

TSV

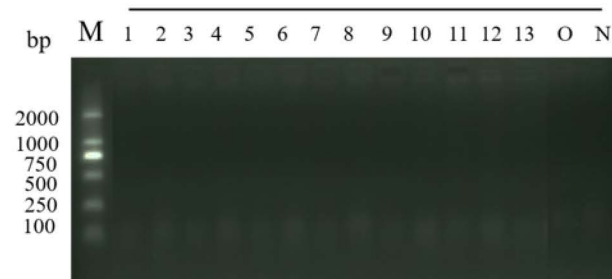

IMNV S1&amp;S2

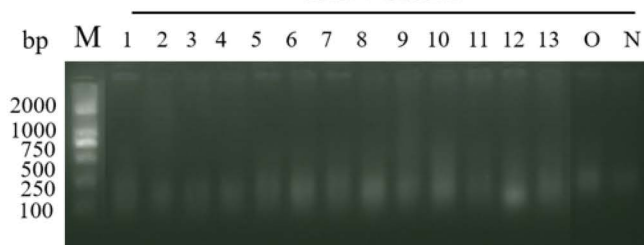

WSSV-S1

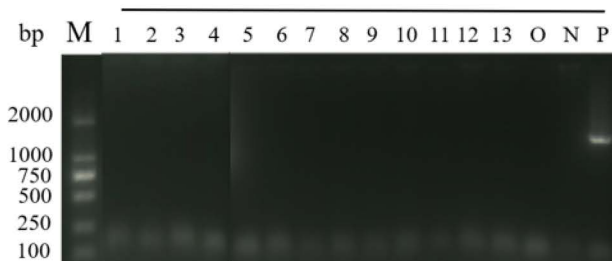

WSSV-S2

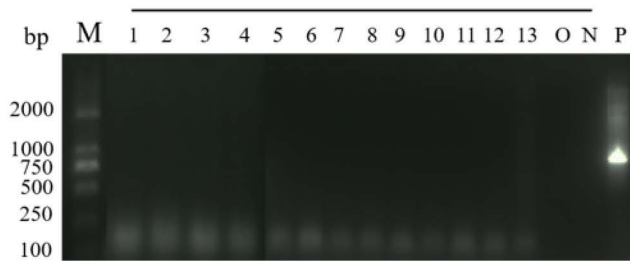 $V_{\text{AHPND}}$  S1&S2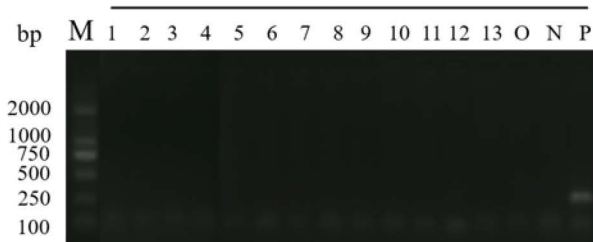

DIV1

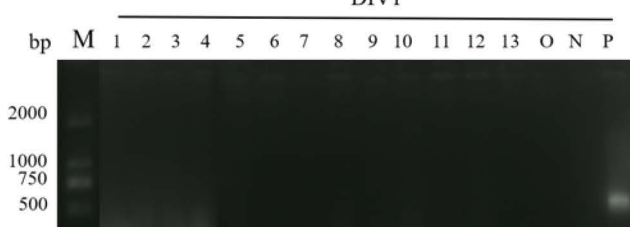

EHP S1&amp;S2

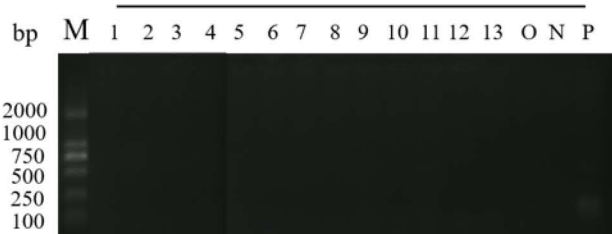

Supplement: FIG S1 [file msystems.00003-21-sf001.pdf]

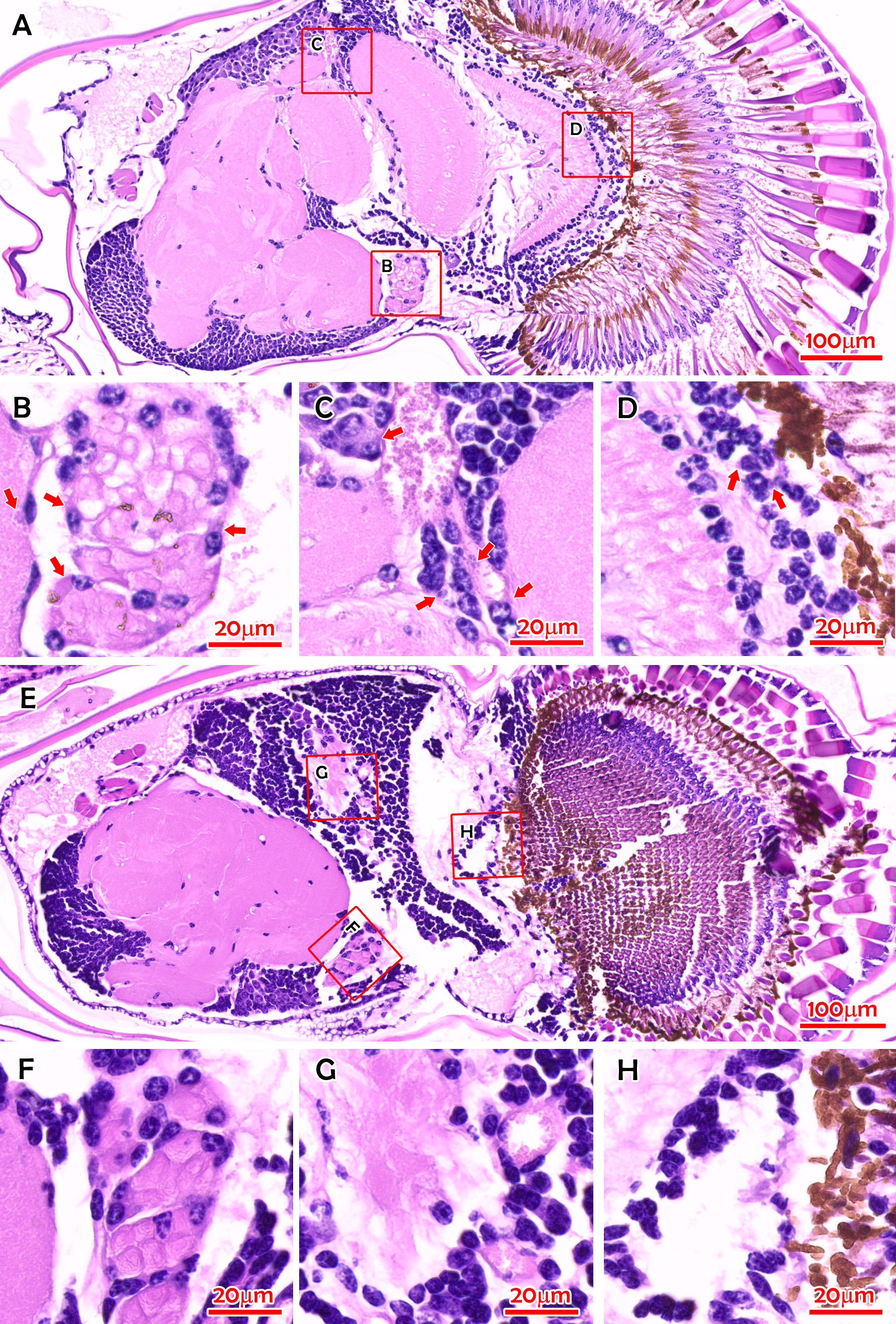

Supplement: FIG S2 [file msystems.00003-21-sf002.jpg]

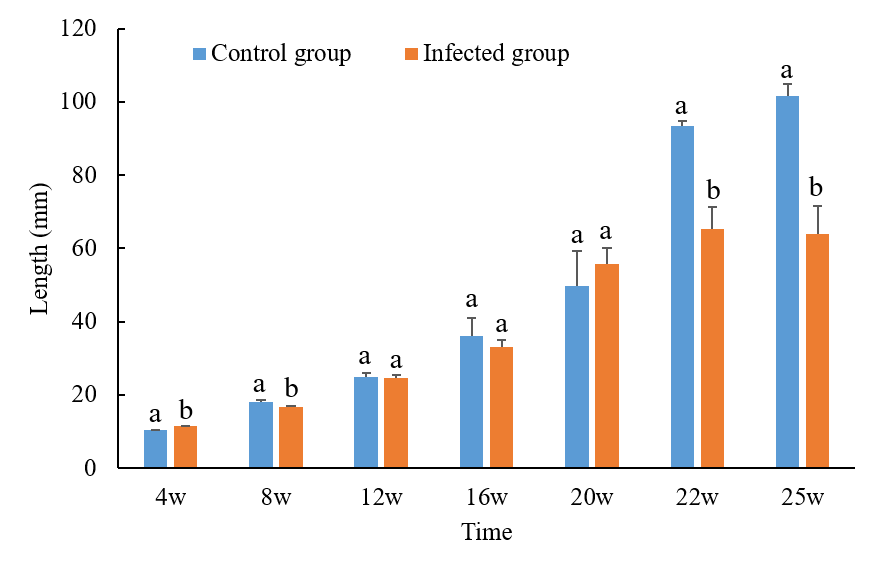

Supplement: FIG S3 [file msystems.00003-21-sf003.tif]

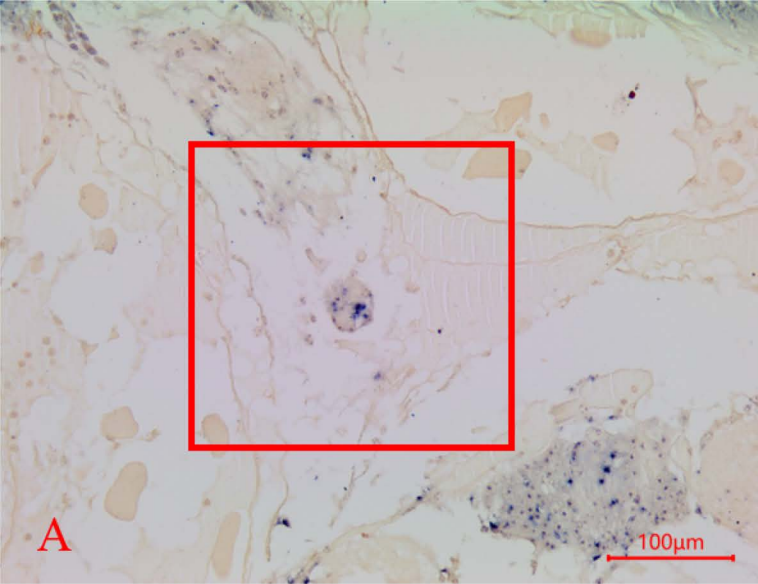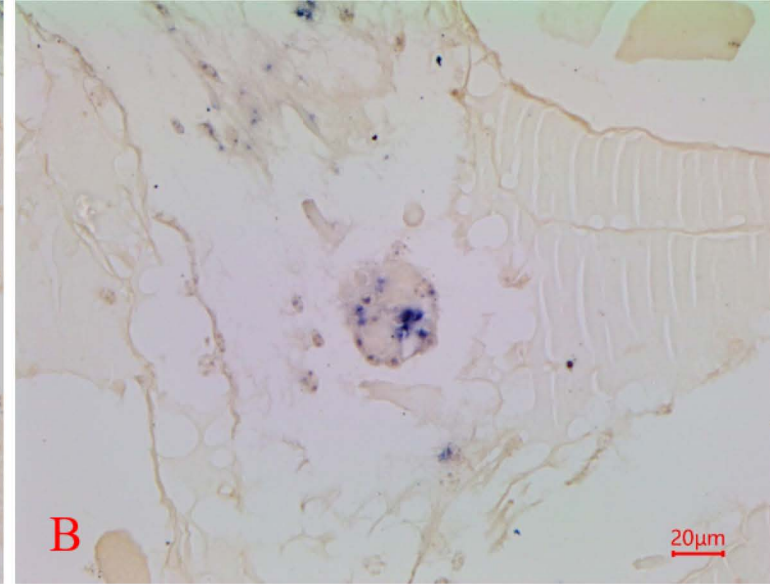

Supplement: FIG S4 [file msystems.00003-21-sf004.pdf]

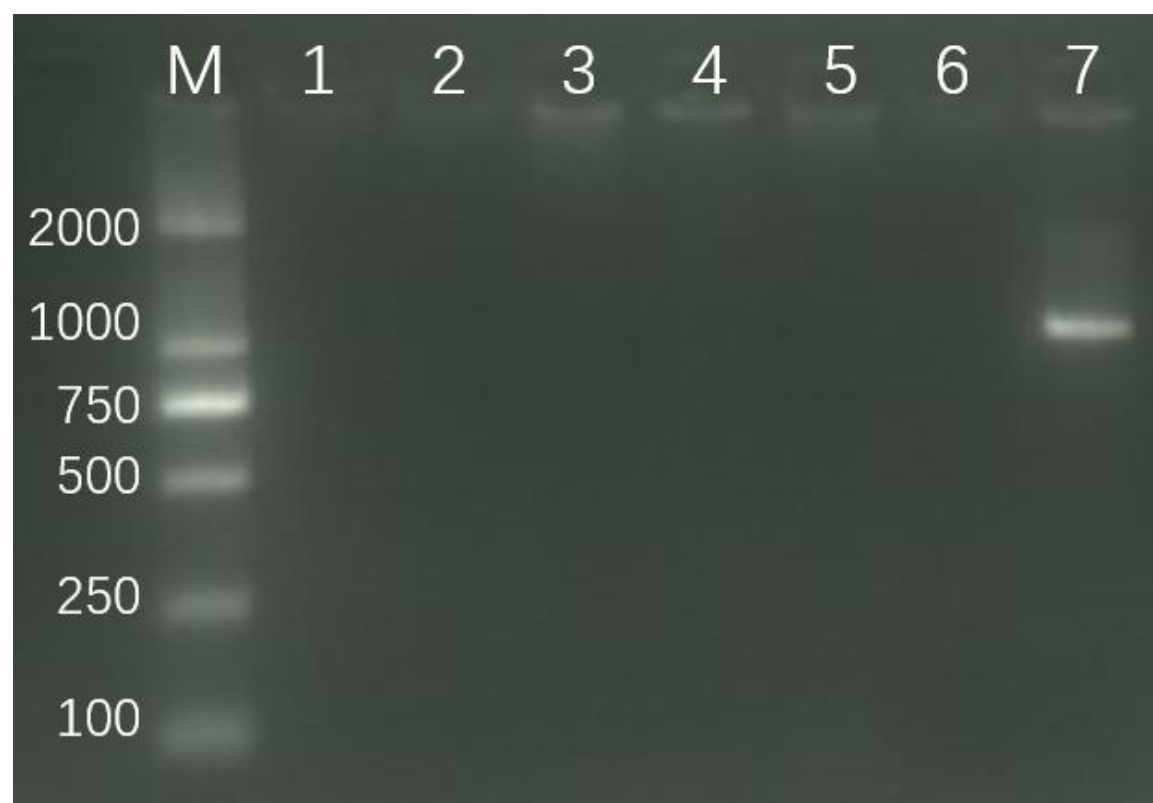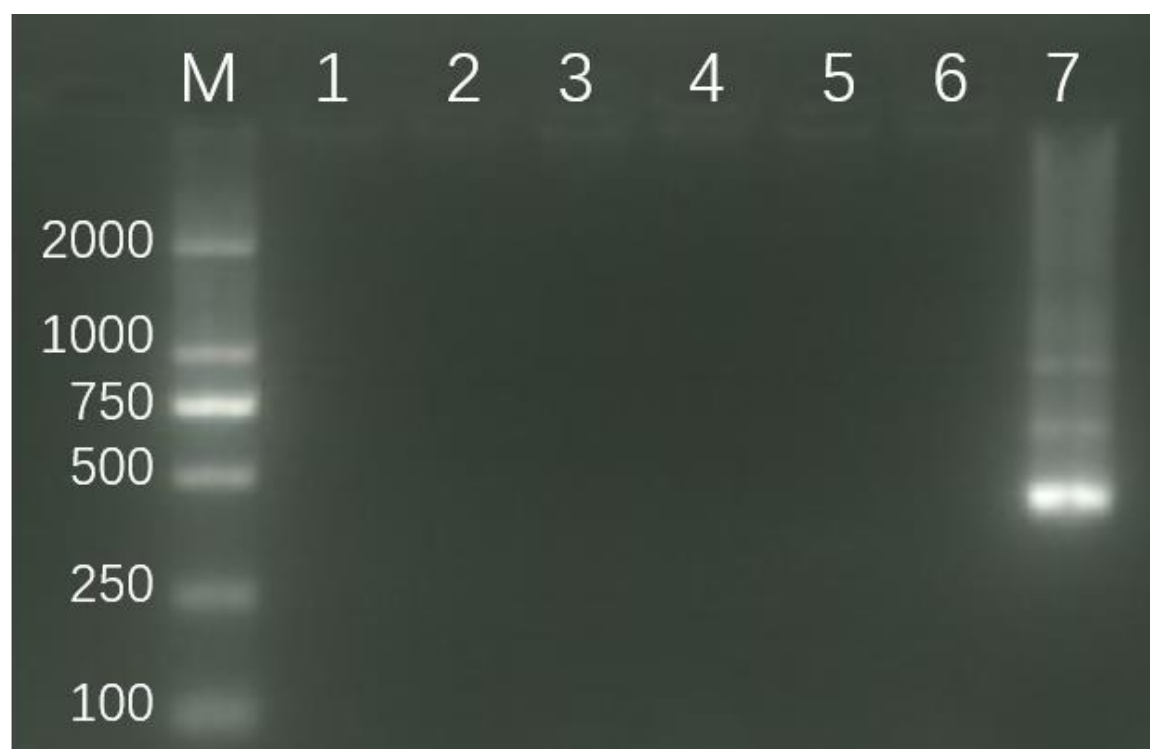

Supplement: FIG S5 [file msystems.00003-21-sf005.pdf]

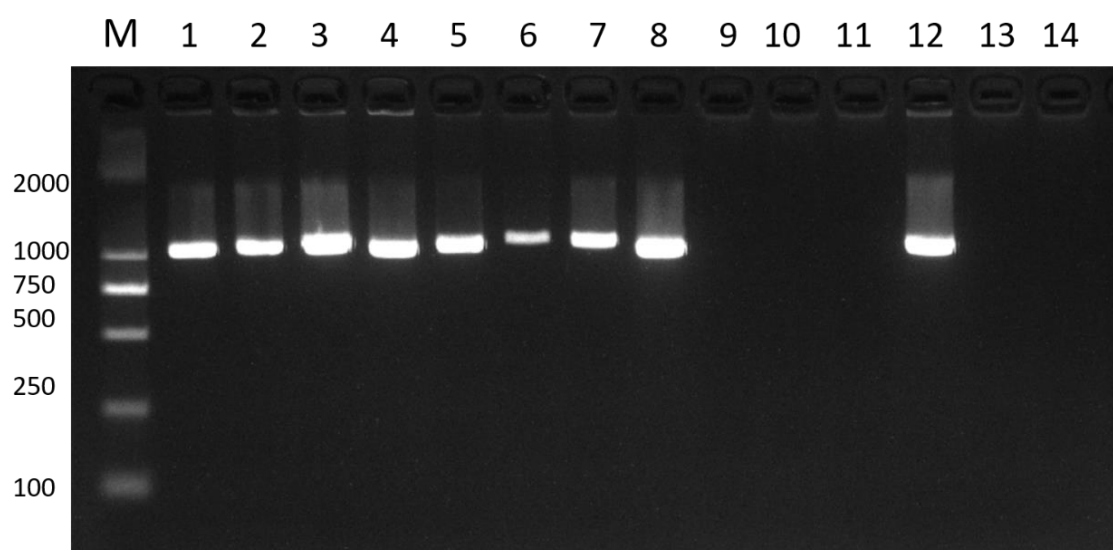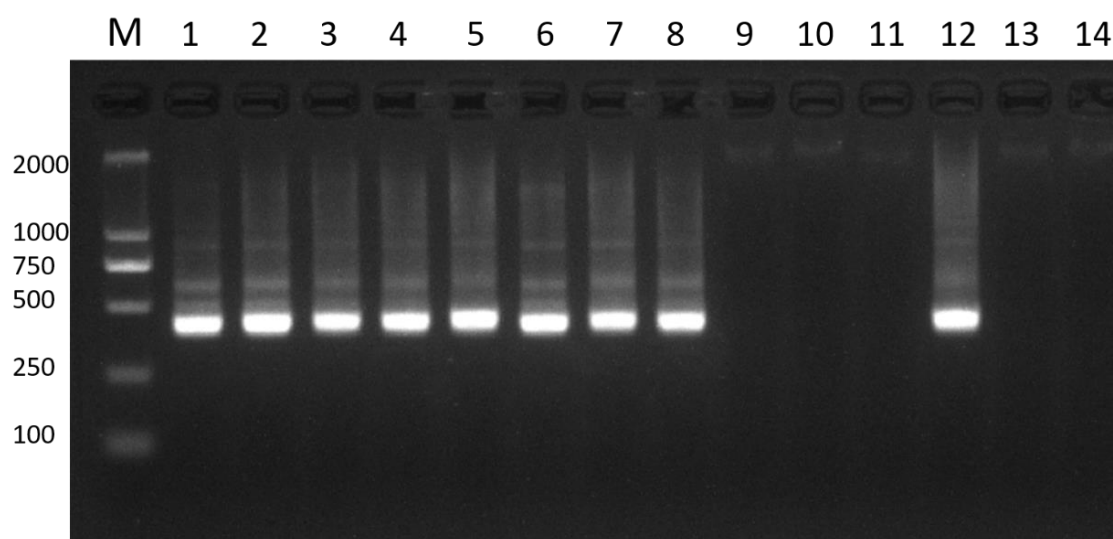

Supplement: FIG S6 [file msystems.00003-21-sf006.pdf]
