# Supplementary material for: A Novel Virus of Flaviviridae Associated with Sexual Precocity in Macrobrachium rosenbergii
Source: mSystems. 2021 Jun 8;6(3):e00003-21. doi: 10.1128/mSystems.00003-21 (PMC8269200; doi:10.1128/mSystems.00003-21)
Supplement: TABLE S1 [file msystems.00003-21-st001.pdf]

Supplementary Table 1 Information of sample marks mentioned in the paper

Mark	Sample code	Organism	Collection date	Source	Gross signs	BioProject accession
NF0	20180724001	*M. rosenbergii*	24 Jul 2018	sample from farm	Healthy	NA
DF0	20180724002	*M. rosenbergii*	24 Jul 2018	sample from farm	IPS	NA
NP	normal preparation from NF0	NA	NA	NA	NA	PRJNA675895
DP	viral preparation from DF0	NA	NA	NA	NA	PRJNA675895
NC1	20190123001-O	*M. rosenbergii*	23 Jan 2019	sample from challenge study	Healthy	PRJNA675895
NC2	20190123003-O	*M. rosenbergii*	23 Jan 2019	sample from challenge study	Healthy	PRJNA675895
DC1	20190123006-O	*M. rosenbergii*	23 Jan 2019	sample from challenge study	IPS	PRJNA675895
DC2	20190123006-X	*M. rosenbergii*	23 Jan 2019	sample from challenge study	IPS	PRJNA675895
DC3	20190123009-O	*M. rosenbergii*	23 Jan 2019	sample from challenge study	IPS	PRJNA675895
DC4	20190123011-O	*M. rosenbergii*	23 Jan 2019	sample from challenge study	IPS	PRJNA675895
DF1	20190602H1H2H3	*M. rosenbergii*	2 Jun 2019	sample from farm	IPS	PRJNA675895
DF2	20190602S1S2	*M. rosenbergii*	2 Jun 2019	sample from farm	IPS	PRJNA675895
DF3	20190602S3S4	*M. rosenbergii*	2 Jun 2019	sample from farm	IPS	PRJNA675895
DF4	20200821010	*M. rosenbergii*	21 Aug 2020	sample from farm	IPS	NA
DF5	20180603013	*M. rosenbergii*	13 Jun 2018	sample from farm	IPS	NA
0929006	20200929006	*M. rosenbergii*	29 Sep 2020	sample from farm	Healthy	NA
0929036	20200929036	*M. rosenbergii*	29 Sep 2020	sample from farm	IPS	NA
0929037	20200929037	*M. rosenbergii*	20 Sep 2020	Sample from farm	IPS	NA
0821006	20180821006	*M. rosenbergii*	21 Aug 2018	sample from challenge study	Healthy	NA
0821007	20180821007	*M. rosenbergii*	21 Aug 2018	sample from challenge study	IPS	NA

IPS means iron prawn syndrome. NA means not applicable.
