# Supplementary material for: A Novel Virus of Flaviviridae Associated with Sexual Precocity in Macrobrachium rosenbergii
Source: mSystems. 2021 Jun 8;6(3):e00003-21. doi: 10.1128/mSystems.00003-21 (PMC8269200; doi:10.1128/mSystems.00003-21)
Supplement: TABLE S2 [file msystems.00003-21-st002.pdf]

Supplementary Table 2 Nested RT-PCR detection of IPV in *M. rosenbergii*

No.	Species	IPV_RT-PCR1	IPV_RT-PCR2	gross signs	Farm
1	*M. rosenbergii*	P	P	IPS	Farm 1
2	*M. rosenbergii*	P	P	IPS	Farm 1
3	*M. rosenbergii*	N	P	IPS	Farm 1
4	*M. rosenbergii*	P	P	IPS	Farm 1
5	*M. rosenbergii*	N	P	IPS	Farm 1
6	*M. rosenbergii*	N	P	IPS	Farm 1
7	*M. rosenbergii*	N	P	IPS	Farm 1
8	*M. rosenbergii*	P	P	IPS	Farm 2
9	*M. rosenbergii*	P	P	IPS	Farm 2
10	*M. rosenbergii*	P	P	IPS	Farm 2
11	*M. rosenbergii*	P	P	IPS	Farm 2
12	*M. rosenbergii*	P	P	IPS	Farm 3
13	*M. rosenbergii*	P	P	IPS	Farm 3
14	*M. rosenbergii*	P	P	IPS	Farm 3
15	*M. rosenbergii*	P	P	IPS	Farm 4
16	*M. rosenbergii*	P	P	IPS	Farm 4
17	*M. rosenbergii*	P	P	IPS	Farm 5
18	*M. rosenbergii*	P	P	IPS	Farm 5
19	*M. rosenbergii*	P	P	IPS	Farm 5
20	*M. rosenbergii*	P	P	IPS	Farm 6
21	*M. rosenbergii*	P	P	IPS	Farm 6
22	*M. rosenbergii*	P	P	IPS	Farm 6
23	*M. rosenbergii*	P	P	IPS	Farm 6
24	*M. rosenbergii*	P	P	IPS	Farm 6
25	*M. rosenbergii*	N	P	Unknown	Farm 7
26	*M. rosenbergii*	N	P	Unknown	Farm 7
27	*M. rosenbergii*	N	P	Unknown	Farm 7
28	*M. rosenbergii*	N	P	Unknown	Farm 7
29	*M. rosenbergii*	N	N	Unknown	Farm 8
30	*M. rosenbergii*	N	N	Healthy	Farm 9
31	*M. rosenbergii*	N	N	Healthy	Farm 9
32	*M. rosenbergii*	N	N	Healthy	Farm 9
33	*M. rosenbergii*	N	N	Healthy	Farm 9
34	*M. rosenbergii*	N	P	Unknown	Farm 10
35	*M. rosenbergii*	N	P	Unknown	Farm 10
36	*M. rosenbergii*	N	P	Unknown	Farm 10
37	*M. rosenbergii*	N	P	Unknown	Farm 10
38	*M. rosenbergii*	N	N	Unknown	Farm 11
39	*M. rosenbergii*	P	P	IPS	Farm 12
40	*M. rosenbergii*	P	P	IPS	Farm 12
41	*M. rosenbergii*	P	P	IPS	Farm 12
42	*M. rosenbergii*	N	P	IPS	Farm 12

43	*M. rosenbergii*	P	P	IPS	Farm 13
44	*M. rosenbergii*	N	P	IPS	Farm 13
45	*M. rosenbergii*	P	P	IPS	Farm 13
46	*M. rosenbergii*	P	P	IPS	Farm 13
47	*M. rosenbergii*	N	P	Unknown	Farm 14
48	*M. rosenbergii*	N	P	Unknown	Farm 14
49	*M. rosenbergii*	N	P	Unknown	Farm 14
50	*M. rosenbergii*	N	P	Unknown	Farm 14
51	*M. rosenbergii*	N	P	Unknown	Farm 15
52	*M. rosenbergii*	N	P	Unknown	Farm 15
53	*M. rosenbergii*	N	N	Unknown	Farm 15
54	*M. rosenbergii*	N	P	Unknown	Farm 15
55	*M. rosenbergii*	P	P	IPS	Farm 16
56	*M. rosenbergii*	P	P	IPS	Farm 16
57	*M. rosenbergii*	P	P	IPS	Farm 16
58	*M. rosenbergii*	P	P	IPS	Farm 16
59	*M. rosenbergii*	N	N	Unknown	Farm 17
60	*M. rosenbergii*	N	P	Unknown	Farm 17
61	*M. rosenbergii*	N	P	Unknown	Farm 17
62	*M. rosenbergii*	N	P	Unknown	Farm 17
63	*M. rosenbergii*	N	P	Unknown	Farm 18
64	*M. rosenbergii*	N	P	Unknown	Farm 18
65	*M. rosenbergii*	N	P	Unknown	Farm 18
66	*M. rosenbergii*	N	P	Unknown	Farm 18
67	*M. rosenbergii*	P	P	IPS	Farm 19
68	*M. rosenbergii*	P	P	IPS	Farm 19
69	*M. rosenbergii*	P	P	IPS	Farm 19
70	*M. rosenbergii*	P	P	IPS	Farm 19
71	*M. rosenbergii*	P	P	IPS	Farm 20
72	*M. rosenbergii*	P	P	IPS	Farm 20
73	*M. rosenbergii*	P	P	IPS	Farm 20
74	*M. rosenbergii*	P	P	IPS	Farm 20
75	*M. rosenbergii*	N	N	Unknown	Farm 21
76	*M. rosenbergii*	N	N	Unknown	Farm 21
77	*M. rosenbergii*	N	P	Unknown	Farm 21
78	*M. rosenbergii*	N	P	Unknown	Farm 21
79	*M. rosenbergii*	N	P	Unknown	Farm 22
80	*M. rosenbergii*	N	P	Unknown	Farm 22
81	*M. rosenbergii*	N	P	Unknown	Farm 22
82	*M. rosenbergii*	N	P	Unknown	Farm 22
83	*M. rosenbergii*	P	P	IPS	Farm 23
84	*M. rosenbergii*	P	P	IPS	Farm 23
85	*M. rosenbergii*	P	P	IPS	Farm 23
86	*M. rosenbergii*	P	P	IPS	Farm 23

87	*M. rosenbergii*	P	P	IPS	Farm 24
88	*M. rosenbergii*	P	P	IPS	Farm 24
89	*M. rosenbergii*	P	P	IPS	Farm 24
90	*M. rosenbergii*	P	P	IPS	Farm 24
91	*M. rosenbergii*	N	N	Unknown	Farm 25
92	*M. rosenbergii*	N	P	Unknown	Farm 25
93	*M. rosenbergii*	P	P	IPS	Farm 26
94	*M. rosenbergii*	P	P	IPS	Farm 26
95	*M. rosenbergii*	P	P	IPS	Farm 26
96	*M. rosenbergii*	P	P	IPS	Farm 26
97	*M. rosenbergii*	P	P	IPS	Farm 27
98	*M. rosenbergii*	P	P	IPS	Farm 27
99	*M. rosenbergii*	P	P	IPS	Farm 27
100	*M. rosenbergii*	N	N	Healthy	Farm 28
101	*M. rosenbergii*	N	N	Healthy	Farm 28
102	*M. rosenbergii*	P	P	IPS	Farm 29
103	*M. rosenbergii*	P	P	IPS	Farm 29
104	*M. rosenbergii*	P	P	IPS	Farm 29
105	*M. rosenbergii*	P	P	IPS	Farm 29
106	*M. rosenbergii*	P	P	IPS	Farm 29
107	*M. rosenbergii*	P	P	IPS	Farm 30
108	*M. rosenbergii*	P	P	IPS	Farm 30
109	*M. rosenbergii*	P	P	IPS	Farm 30
110	*M. rosenbergii*	P	P	IPS	Farm 30
111	*M. rosenbergii*	P	P	IPS	Farm 30
112	*M. rosenbergii*	P	P	IPS	Farm 31
113	*M. rosenbergii*	P	P	IPS	Farm 31
114	*M. rosenbergii*	P	P	IPS	Farm 31
115	*M. rosenbergii*	P	P	IPS	Farm 31
116	*M. rosenbergii*	N	N	Unknown	Farm 32
117	*M. rosenbergii*	N	N	Healthy	Farm 33
118	*M. rosenbergii*	N	N	Healthy	Farm 33
119	*M. rosenbergii*	N	N	Healthy	Farm 33
120	*M. rosenbergii*	N	N	Healthy	Farm 33
121	*M. rosenbergii*	N	N	Healthy	Farm 33
122	*M. rosenbergii*	N	N	Healthy	Farm 33
123	*M. rosenbergii*	N	N	Healthy	Farm 33
124	*M. rosenbergii*	N	N	Healthy	Farm 33
125	*M. rosenbergii*	N	N	Healthy	Farm 33
126	*M. rosenbergii*	N	N	Healthy	Farm 33
127	*M. rosenbergii*	N	N	Healthy	Farm 33
128	*M. rosenbergii*	N	N	Healthy	Farm 33
129	*M. rosenbergii*	N	N	Healthy	Farm 33
130	*M. rosenbergii*	N	N	Healthy	Farm 33

131	*M. rosenbergii*	N	N	Healthy	Farm 33
132	*M. rosenbergii*	N	N	Healthy	Farm 33
133	*M. rosenbergii*	P	P	Unknown	Farm 34
134	*M. rosenbergii*	N	P	Unknown	Farm 34
135	*M. rosenbergii*	P	P	Unknown	Farm 35
136	*M. rosenbergii*	P	P	Unknown	Farm 35
137	*M. rosenbergii*	P	P	Unknown	Farm 35
138	*M. rosenbergii*	N	P	Unknown	Farm 35
139	*M. rosenbergii*	N	N	Healthy	Farm 36
140	*M. rosenbergii*	N	N	Healthy	Farm 36
141	*M. rosenbergii*	N	N	Healthy	Farm 36
142	*M. rosenbergii*	N	N	Healthy	Farm 36

‘Unknown’ means that the symptom is not clear when the sample was collected.

IPS means iron prawn syndrome.
