# Supplementary material for: A Novel Virus of Flaviviridae Associated with Sexual Precocity in Macrobrachium rosenbergii
Source: mSystems. 2021 Jun 8;6(3):e00003-21. doi: 10.1128/mSystems.00003-21 (PMC8269200; doi:10.1128/mSystems.00003-21)
Supplement: TABLE S3 [file msystems.00003-21-st003.pdf]

Supplementary Table 3 Nucleotide sequences of PCR primers used in this study.

Primers	5'-3'
IPV-F1	CTGTTAAACCCCAGGTGGAGG
IPV-R1	AGGCTTCAATCTTAAAGGAGGCTA
IPV-F2	CACCCAGAGCACCCACAAG
IPV-R2	TGGGATGTGTGGTACATGTTTAAGT
IPV-F3	CTTTGCGGTTGAGGGCTG
IPV-R3	TGTGCTGCTAGTGAGCAGAGGT
IPV-F4	TTCTGGCATATCATATGAGGAAAAG
IPV-R4	CTGGACGTCACTGAAGGAAGTCT
IPV-F5	AACTCTCATACTTATACCAAGAAGGAACT
IPV-R5	GTGCAGAAAACCCGAACCAT
IPV-F6	TCTATGATCTGTGACTCTTTCTTGGTT
IPV-R6	CATTGAGGTTTATCGGAGGCA
IPV-F7	AACCTGAGAGGCTCTGAGACAAGTA
IPV-R7	GCTATAATAGTTCTTGCAACCTTTGTCT
IPV-F8	TGGAGGTGGACTTGCTTGTTCT
IPV-R8	GCACTATTGAGTGAACAAGATGGAA
IPV-F9	AACGAAGAGAGTGACCCCCAA
IPV-R9	TCCAAATTGTACACAAGGGCAC
IPV-F10	ATACCCCAAGACTCCACAGAAAG
IPV-R10	GTCTGCCCTAGAGGTCTGTGCT
IPV-F11	TGATGGTAGTGGGCCTTGTTATAT
IPV-R11	AATGCTAAATGTTATGAAGGGAAAGTA
IPV-F12	AGTGACTCGTACAGTCGGCCTAA
IPV-R12	TGGGTGTCATATTACTCTCCCTTACTA
IPV-F13	ACAACCATCCATGGGAATTGA
IPV-R13	GATGAACTAGCGGGGTGTTACTACT
IPV-F14	ACTGACGGAGGAACTTCAAGG
IPV-R14	CCAACTAACAGGGTCTTTTTCAA
5’adaptor	GCTGTCAACGATACGCTACGTAACGGCATGACAGTGGGIIGGGIIGGGIIG
3’adaptor	GCTGTCAACGATACGCTACGTAACGGCATGACAGTGTTTTTTTTTTTTTTTTTT
5.3’outer	GCTGTCAACGATACGCTACGTAAC
5.3’inner	GCTACGTAACGGCATGACAGTG
IPV-OUT-NF1	TGAGACTCCCTTCAAGGCCTATGTATTAGAGT
IPV-OUT-NF2	GTAAGACCTTCGGCTAATTGGCCACAGA
IPV-OUT-NR2	AAATCATAATGGTGCCTGTAAAGGTGTGACT
IPV-OUT-NR1	CCTGTTAGTTGGGTTCTAAAGGGAAGTGCT
IPV-OUT-NRT2	CTTACAGCTTCACCCACATTTTG
IPV-OUT-NRT1	CGGTAATGAGAGTCACCTGGTT
