# Supplementary material for: A Novel Virus of Flaviviridae Associated with Sexual Precocity in Macrobrachium rosenbergii
Source: mSystems. 2021 Jun 8;6(3):e00003-21. doi: 10.1128/mSystems.00003-21 (PMC8269200; doi:10.1128/mSystems.00003-21)
Supplement: TABLE S4 [file msystems.00003-21-st004.docx]

| Supplementary Table 4 Information of sequences used in Fig. 4. | | | |
| --- | --- | --- | --- |
| Virus | Abbreviations | NS3_accession no. | RdRp_accession no. |
| Alongshan virus | ALSV | QDO16173 | AXE71873 |
| Alongshan virus | ALSV | QDO16167 | QDO16174 |
| Apoi virus | APOIV | NP_620045 | NP_620045 |
| Beihai barnacle viurs 1 | BHBV | YP_009179226 | YP_009179226 |
| Bole tick virus 4 | BLTV4 | YP_009179221 | YP_009179221 |
| Bovine viral diarrhea virus 1 | BVDV-1 | NP_040937 | NP_040937 |
| Cell fusing agent virus | CFAV | YP_009259257 | YP_009259257 |
| Crangon crangon flavivirus | CcFV | QCH00713 | QCH00713 |
| Dengue virus 1 | DENV1 | NP_059433 | NP_059433 |
| Equine hepacivirus | EHcV | YP_009058898 | YP_009058898 |
| Firefly squid flavivirus | FfSFV | QCH00715 | QCH00714 |
| Gamboa mosquito virus | GMV | YP_009179224 | YP_009179224 |
| Gammarus chevreuxi flavivirus | GcFV | QCH00712 | QCH00712 |
| Gammarus pulex flavivirus | GpFV | QCH00716 | QCH00716 |
| Gentian Kobu-sho-associated virus | GKaV | YP_007438864 | YP_007438864 |
| Hepatitis C virus | HCV | NP_671491 | NP_671491 |
| Hepatitis GB virus B | GBV-B | NP_056931 | NP_056931 |
| Japanese encephalitis virus | JEV | NP_059434 | NP_059434 |
| Jingmen tick virus | JMTV | YP_009030000 | YP_009029999 |
| Mosquito flavivirus K928 | MosV | YP_007877501 | YP_007877501 |
| Norway rat pestivirus | NrPV | YP_009109567 | YP_009109567 |
| Pegivirus A | GBV-A | NP_045010 | NP_045010 |
| Pegivirus B | GBV-D | YP_009256194 | YP_009256194 |
| Rodent hepacivirus | RHV | YP_007905733 | YP_007905733 |
| Sabethes flavivirus | SbFV | AZB73874 | AZB73874 |
| Sanxia water strider virus 6 | SXWSV6 | YP_009179218 | YP_009179218 |
| Shayang fly virus 4 | SYFV4 | YP_009179225 | YP_009179225 |
| Shayang spider virus 4 | SYSV4 | YP_009179219 | YP_009179219 |
| Shuangao insect virus 7 | SAIV7 | YP_009179402 | YP_009179401 |
| Shuangao lacewing virus 2 | SALV2 | YP_009179223 | YP_009179223 |
| Southern pygmy squid flavivirus | StPSFV | QCH00711 | QCH00711 |
| Soybean cyst nematode virus 5 | SbCNV-5 | YP_009028573 | YP_009028573 |
| Tacheng tick virus 8 | TCTV8 | YP_009179217 | YP_009179217 |
| Tamana bat virus | TABV | NP_658908 | NP_658908 |
| Tick-borne encephalitis virus | TBEV | NP_043135 | NP_043135 |
| Wenling shark virus | WLSV | YP_009179227 | YP_009179227 |
| Wenzhou shark flavivirus | WZSFV | MK473876 | AVM87250 |
| West Nile virus | WNV | NP_041724 | NP_041724 |
| Wuhan aphid virus 1 | WHAV1 | YP_009179389 | YP_009179388 |
| Wuhan aphid virus 2 | WHAV2 | YP_009179379 | YP_009179378 |
| Wuhan centipede virus | WHCev | YP_009254745 | YP_009254745 |
| Wuhan cricket virus | WHCV | YP_009179400 | YP_009179405 |
| Wuhan flea virus | WHFV | YP_009179404 | YP_009179403 |
| Xingshan cricket virus | XSCV | YP_009179220 | YP_009179220 |
| Xinzhou spider virus 2 | XZSV2 | YP_009179222 | YP_009179222 |
| Xinzhou spider virus 3 | XZSV3 | YP_009254746 | YP_009254746 |
| Yanggou tick virus | YGTV | QBQ65082 | QBQ65056 |
| Yellow fever virus | YFV | NP_041726 | NP_041726 |
